# N-terminally acetylated Met11-Tau: a new pathological truncated Tau species with functional relevance in Alzheimer's disease

**DOI:** 10.1186/s40035-026-00550-8

**Published:** 2026-04-27

**Authors:** Sarah Guedjdal, Coline Leghay, Maxime Derisbourg, Sabiha Eddarkaoui, Simon Lecerf, Florian Vermon, Raphaelle Caillierez, Séverine Begard, Claire Regost, Charlotte Laloux, Paulo J. da Costa, Kevin Carvalho, Giovanni Chiappetta, Yann Verdier, Valérie Buée-Scherrer, Vincent Deramecourt, Susanna Schraen, David Blum, Franck Martin, Luc Buée, Malika Hamdane

**Affiliations:** 1 Univ. Lille, Inserm, CHU Lille, Lille Neuroscience & Cognition, Lille, France; 2 Univ. Lille, CNRS, Inserm, CHU Lille, Institut Pasteur de Lille, US 41 - UAR 2014 - PLBS, Lille, France; 3 CNRS UPR9002, Université de Strasbourg, Institut de Biologie Moléculaire et Cellulaire, Architecture et Réactivité de l’ARN, Strasbourg, France; 4 CNRS UAR2051, SMBP, ESPCI Paris, PSL University, Paris, France

**Keywords:** Tau modifications, Tau truncation, Neurofibrillary degeneration, Tauopathies, Passive immunotherapy

## Abstract

**Background:**

Tauopathies are a group of neurodegenerative diseases, including Alzheimer’s disease (AD), characterized by progressive accumulation of pathological Tau proteins. Among the diverse Tau species, truncated variants are emerging as key contributors, yet their identity remains elusive, particularly for the N-terminal truncated ones. The present study identifies and characterizes a novel N-terminally truncated and N-alpha-acetylated form of the Tau protein, named AcMet11-Tau.

**Methods:**

We identified AcMet11-Tau by further analyses of previous proteomic data (capillary liquid chromatography-tandem mass spectrometry). We developed a monoclonal antibody, termed 2H2D11, by hybridoma method. The specificity of 2H2D11 was validated by ELISA, Western blot and immunohistochemistry. Expression of AcMet11-Tau in transgenic mouse model of Tau pathology and postmortem brain tissues was analyzed by ELISA and/or immunohistochemistry. Overexpression of AcMet11-Tau in the mouse brain was achieved by stereotaxic injections of lentiviral vectors carrying the coding sequence in the hippocampus. To neutralize AcMet11-Tau, transgenic mice received repeated intraperitoneal immunizations with either 2H2D11 or control antibody. The effects on Tau pathology were assessed by immunohistochemistry, qPCR, and behavioral assays.

**Results:**

Using 2H2D11, the newly developed antibody specifically targeting the AcMet11-Tau variant, we demonstrated that this species accumulated early in degenerating neurons in both transgenic mouse models of AD-related Tau pathology and post-mortem brain tissues from AD patients. Importantly, in vivo functional experiments revealed that expression of this truncated Tau species exacerbated Tau pathology in the transgenic mice, whereas targeted immunotherapeutic with the specific 2H2D11 antibody significantly reduced pathological Tau accumulation and prevented associated memory impairments.

**Conclusion:**

These findings position this newly identified Tau variant as a marker of neurofibrillary degeneration and a Tau species that contributes to disease-associated pathological processes, supporting its potential as a therapeutic target in Tau-related disorders, notably AD.

**Supplementary Information:**

The online version contains supplementary material available at 10.1186/s40035-026-00550-8.

## Introduction

Tau is a microtubule-associated protein primarily expressed in neurons, where it plays, among others, a fundamental role in microtubule stabilization and intracellular transport. In the adult human brain, six Tau isoforms are produced from a single gene by alternative splicing of exons 2, 3 and 10 [1]. Tauopathies are a group of neurodegenerative disorders, including Alzheimer’s disease (AD), progressive supranuclear palsy (PSP), and frontotemporal lobar degeneration, that are characterized by pathological modifications of the Tau protein such as hyperphosphorylation, truncation and aggregation [2]. AD is the most common tauopathy and the leading cause of dementia. A key neuropathological hallmark of AD is the neurofibrillary degeneration (NFD), which consists in intracellular aggregates of Tau proteins bearing abnormal post-translational modifications. Neuropathological and biochemical studies have consistently shown that the progression of NFD in cortical brain areas strongly correlates with the cognitive decline in AD, highlighting Tau as a central player in AD pathology [3–5]. However, the precise mechanisms underlying the formation and regional spread of NFD remain incompletely understood. Key unresolved questions include identification of the particular forms of Tau that confer toxicity and the mechanisms by which these species promote neurodegeneration. Among the diverse Tau species observed in AD brains, truncated variants are increasingly recognized as potential pathogenic contributors [6–10]. Using an optimized proteomic approach, we previously identified several novel N-terminally truncated Tau species from the AD human brain tissue, including a variant starting at the residue methionine11 (Met11-Tau) [11]. This Tau variant is particularly interesting because Met11 is located in exon 1, the N-terminal region common to all Tau protein isoforms. Although the functional roles of exon 1 have not been extensively investigated, emerging evidence suggests that modifications in this region may influence Tau functions and contribute to Tau pathology [11–14]. The N-terminal domain of Tau is thought to play a structural role, notably through its involvement in the formation of a compact “paperclip” conformation of Tau protein, which results from intramolecular interaction between N-terminal and C-terminal domains [15–17]. Consequently, the loss of the outermost N-terminus of Tau, as encountered in Met11-Tau, might have significant functional and pathological implications. Notably, our proteomic data revealed that Met11-Tau is present in an N-terminally acetylated form (AcMet11-Tau). N-terminal acetylation is among the most common protein modifications in eukaryotes, affecting approximately 80% of human proteins [18, 19]. This irreversible co-translational modification involves the transfer of an acetyl group from acetyl-coenzyme A to the α-amino group of a nascent polypeptide’s N-terminus, catalyzed by Nα-terminal acetyltransferases [20]. The functional implications of N-terminal acetylation are diverse and significant, including the regulation of protein stability and conformation. Moreover, dysregulation of N-terminal acetylation has been involved in a range of human diseases, including cancer and neurodegenerative disorders, underscoring its critical role in maintaining cellular homeostasis [21, 22]. Given the established functional roles of Tau truncation and N-terminal acetylation, elucidating the contribution of the newly identified AcMet11-Tau species to AD-related Tau pathology is of considerable interest. To address this issue, we generated a monoclonal antibody with high specificity for the AcMet11-Tau variant. Through studies in a transgenic mouse model of tauopathy and analyses of postmortem human brain tissues, we demonstrated the presence of AcMet11-Tau in neurons displaying NFD at early pathological stages. Notably, immunotherapy targeting AcMet11-Tau ameliorated both pathological burden and memory deficits in Tau transgenic mice. Collectively, these findings identify AcMet11-Tau as a previously unrecognized, early pathological Tau species with physiopathological and therapeutic implications for AD.

## Materials and methods

### Study design

The primary aim of this study was to characterize AcMet11-Tau, a previously overlooked truncated Tau protein that we identified as an N-α-acetylated form in human brain by proteomic approach. To address this issue, it was necessary to get first a specific monoclonal antibody with no cross reactivity with full-length Tau proteins. This immunological tool was then used to examine brain tissue samples from a transgenic mouse model of Tau pathology and human brains. Data analysis indicated that AcMet11-Tau may be implicated in Tau pathology development. To address this hypothesis, we subsequently performed functional studies in the transgenic mice.

Two lines of Thy-Tau transgenic mice were used in this study. The Thy-Tau22 line is a well-established model for investigating modifiers of Tau pathology and their impact on memory. These mice develop progressive hippocampal Tau pathology before 3 months of age, with memory deficits developing from 6–7 months. The Thy-Tau22 model recapitulates key aspects of early and progressive Tau pathology, particularly in the hippocampus, a brain region critically affected during the initial stages of AD. This model is therefore especially well-suited for investigating factors that modulate early Tau aggregation and associated functional deficits, such as hippocampus-dependent cognitive impairment. While it does not encompass the full spectrum of AD pathology—including widespread cortical involvement and extensive neuronal loss—its focus on early pathological events provides a valuable framework for dissecting mechanisms relevant to disease initiation and progression. This transgenic line was then used for intraperitoneal (IP) passive immunizations.

The Thy-Tau30 line exhibits a more rapid disease course, with widespread neurofibrillary tangle (NFT) accumulation, axonopathy and severe motor deficits, which can confound memory assessments. Accordingly, this line was used only in experiments that did not involve behavioral testing.

Mice were IP immunized in statistically valid number (*n* = 10–15 per group), based on prior experience with the Thy-Tau22 model. Both male and female mice were included in the immunization groups. Behavioral assessments were performed in males only, while Tau pathology analyses were performed on pooled data from both males and females.

The selection of human brain areas for AcMet11-Tau detection was based on both tissue availability and their relevance to the known spatiotemporal progression of tau pathology. Brain extracts were analyzed by Western blot with antibodies against Total Tau (Tau-Cter) and Tau-pSer396. One patient (#C23, Table S1), whose biochemical profile matched Braak stage III–IV, was reassigned to this group for ELISA analyses despite a neuropathological diagnosis of NFT at Braak stage II.

Additional points related to the study design are presented in other sections of the manuscript or in the supplementary material file.

### Human brain tissue

Frozen brain tissue samples from patients diagnosed with various tauopathies, AD, Pick’s disease (PiD), and PSP, as well as from neurologically healthy individuals (Table S1), were obtained from the Lille NeuroBank collection (Centre de Ressources Biologiques du CHU de Lille). For controls and AD cases, Braak staging was categorized by experienced neuropathologists based on histological analysis and the Braak classification system [23]. Patients were grouped according to the stage of NFD (NFT pathology) as follows: Braak stage 0 (no detectable neurofibrillary lesions), Braak stages I–II (early-stage NFTs), Braak stages III–IV (moderate NFT pathology), and Braak stages V–VI (severe NFT pathology). Braak stages I–III indicate early to moderate neurofibrillary pathology (involving the hippocampus, limbic regions, and subsequently temporal areas), yet this remains insufficient to support a definitive diagnosis of AD. These cases are therefore better described as exhibiting early neurofibrillary pathology or compatible with preclinical AD, rather than confirmed AD. Most individuals in the Braak 0 and I–II groups had no clinical history of cognitive impairment or dementia at the time of death. Some individuals in the Braak III–IV group exhibited mild cognitive symptoms. Patients classified as Braak stage V–VI were selected based on documented clinical histories consistent with typical AD progression. After tissue processing, samples were analyzed by Western blot to confirm NFT pathology and staging [5].

### Mice

For primary neuronal culture, gestating C57Bl6/J mice were purchased from Janvier Laboratories (Le Genest-Saint-Isle, France). Thy-Tau transgenic mouse lines (Thy-Tau22 and Thy-Tau30) of the C57Bl6/J background, which develop NFD and memory deficits with age, were generated by overexpressing a human Tau-412 isoform (1N4R) bearing two pro-aggregative mutations (G272V & P301S) under the control of the Thy1.2 neuronal promoter [24]. Additional description has been provided for the Thy-Tau 22 model [25, 26] and the Thy-Tau 30 model [27]. Non-transgenic wild-type littermates (WT) were used as controls. The animals were housed in a pathogen-free facility as group of 5 in ventilated cages, with ad libitum access to food and water and maintained on a 12 h light/12 h dark cycle and controlled temperature (20–22°C). The animals were maintained in compliance with European standards for the care and use of laboratory animals.

### Tau plasmid and lentiviral vectors (Lvs)

Plasmid vectors carrying cDNAs for full-length Tau (FL-Tau; Tau-412 isoform, Uniprot P10636-7) and Met11-Tau (in the same isoform context as FL-Tau) were generated using the In-Fusion® HD Cloning Kit (Cat. No. 639650, Takara Bio USA, Inc., Mountain View, CA), and PCR primers were designed to clone inserts into the EcoRI site of pcDNA4/TO (Cat. No. V102020, Invitrogen, Carlsbad, CA). Each cDNA fragment was amplified by PCR (DyNAzymeTM EXT DNA polymerase, Cat. No. F-501L, New England Biolabs, Ipswich, MA) from pcDNA3.1-Tau4R [28]. Forward primers were designed to contain the Kozak consensus sequence and are as follows: Tau-412, 5'-CAGTGTGGTGGAATTCGCCACCATGGCTGAGCCCCGCCAGGAGTT-3'; Met11-Tau, 5'-CAGTGTGGTGGAATTCGCCACCATGGAAGATCACGCTGGGACGT-3'. The reverse primer for both amplifications was 5'-GATATCTGCAGAATTCTCACAAACCCTGCTTGGCCAGGGAGGCA-3'. Lvs with neuronal tropism [29], carrying cDNAs for FL-Tau and Met11-Tau, were generated, produced and their titers determined as previously detailed [30] using the HIV-1 capsid protein p24 (Gentaur BVBA, Paris, France). DNA sequencing was carried out for construct validation.

### Tau cell lines

SH-SYSY cells that constitutively express tetracycline repressor [31] were transfected with the Tau plasmid constructs using ExGen500 (Euromedex, France) according to the manufacturer's instructions. Individual stable clones were generated following Zeocin selection (100 ${\upmu}$g/mL), and those that exhibited the weakest basal expression of Tau were selected. The cell lines were maintained in Dulbecco's modified Eagle's medium (DMEM, Gibco, Grand Island, NY), supplemented with 10% fetal calf serum with pyruvate, 2 mM L-glutamine, 50 units/mL penicillin/streptomycin and 1 mM non-essential AA, in a 5% CO_2_ humidified incubator at 37 °C. Tau expression was induced by tetracycline at 1 ${\upmu}$g/mL (Invitrogen). These inducible cell lines were used exclusively to validate antibody specificity in a cellular context.

### Primary neuronal culture and infection with Lvs

Primary cortical neurons were prepared from 15-day-old C57Bl6/J mouse embryos as previously described [32]. Once dissociated and counted, cells were plated in poly-D-lysine (0.5 mg/mL, Sigma-Aldrich, St. Louis, MO)- and laminin (10 µg/mL, Sigma-Aldrich)-coated 6-well plates (800,000 cells per well) and maintained in a 5% CO_2_ humidified incubator at 37 °C.

Lv-based infections were performed on day 11 in vitro, as previously described [32]; 400 ng of Lvs were added per well and 3 days later, the cells were washed once with phosphate-buffered saline (PBS) and recovered in lysis buffer for Western blotting analysis.

### Liquid chromatography-tandem mass spectrometry (LC–MS/MS)

AcMet11-Tau was identified using the experimental approaches previously detailed [11]. Briefly, Tau species were enriched by immunoprecipitation from human occipital cortex (Table S1). Peptides were purified on a capillary reversed-phase column (nano C18 Acclaim PepMap100 A, 75 µm inner diameter, 15 cm length; Cat. No. 164568, Dionex, Sunnyvale, CA) at a constant flow rate of 220 nL/min, with a gradient of 2% to 40% of buffer B in buffer A, for 45 min. Buffer A was a mix of water/acetonitrile/formic acid 98:2:0.1 (*v/v/v*), and buffer B was water/ACN/formic acid 10:90:0.1 (*v/v/v*). Coupling to the mass spectrometer was done using a NanoMate (Advion, Inc., Ithaca, NY; Voltage: + 1.70 kV; Spray Sensing Enabled; Below 10.0 nA or above 2000.0 nA for 5 s; Chip ID: A8E159AK). The MS analysis was performed on a Fourier transform ion cyclotron resonance (FT-ICR) mass spectrometer (LTQ-FT Ultra; ThermoFisher Scientific, San Jose, CA) with the top-seven acquisition method: MS resolution 60,000, mass range 470–2000 Da, followed by MS/MS (LTQ) of the seven most intense peaks, with a dynamic exclusion of 90 s. The raw data were processed using Xcalibur 2.0.7 software. The database search was done using the Mascot search engine (Matrix Science Mascot 2.2.04) on a homemade protein databank containing the 6 human Tau isoforms and some contaminants. Proteome Discoverer 1.3 (ThermoFisher Scientific) and Mascot were used to search data and filter the results. The following parameters were used: MS tolerance 5 ppm; MS/MS tolerance 0.5 Da; semi-tryptic peptides; one missed cleavage allowed; partial modifications: carbamidomethylation (C), oxidation (M), phosphorylation (ST), acetylation (N-term), thiopropionation (N-ter, K). The MS proteomics data have been deposited to the ProteomeXchange Consortium (http://proteomecentral.proteomexchange.org) via the PRIDE [33] partner repository with dataset identifiers PXD072580 and 10.6019/PXD072580.

### Generation of monoclonal antibodies

**Fig. 1:**
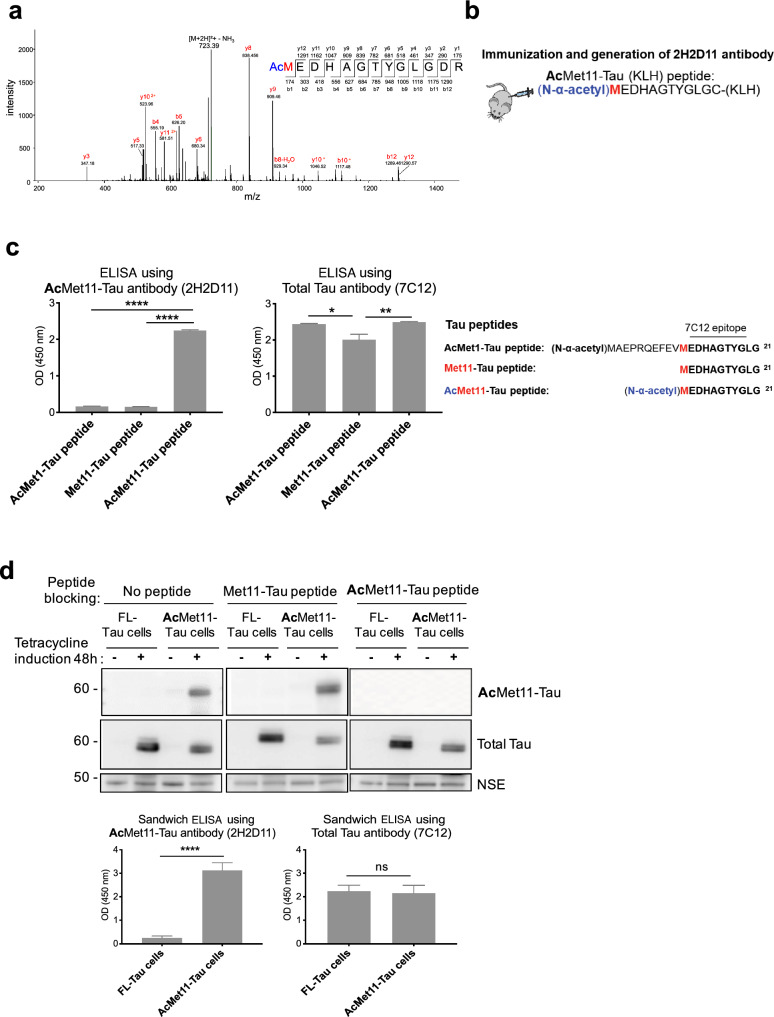
Discovery of AcMet11-Tau protein and validation of specificity of its antibody.** a** MS/MS spectra of N-terminally acetylated peptide Met11-Tau from a human brain (Braak stage III, Table S1). Charge: +2; Monoisotopic m/z: 732.31 Da; MH+: 1463.62; RT: 27.86; identified with Mascot v1.30; ion score = 54; exp value: 3E-003. **b** Representation of 2H2D11 antibody production with the peptide sequence used for immunizations. **c** Histograms indicated representative ELISA OD values obtained by 2H2D11 and 7C12 (Total-Tau) antibodies (mean ± SEM, *n* = 3 independent experiments, *P = 0.0125, **P = 0.0077, ****P < 0.0001, one-way ANOVA followed by Fisher’s LSD post hoc test). Amino acid sequences of the synthetic peptides used in ELISA are shown; the line indicates the 7C12 epitope, according to the longest Tau isoform. **d** Protein extracts from SH-SY5Y inducible cell lines overexpressing either full-length Tau (FL-Tau) or AcMet11-Tau, after 48 h of tetracycline treatment, were analysed by: (top) Western blot using 2H2D11 antibody pre-adsorbed or not with Tau peptides; membranes were re-probed with Total-Tau antibody (Tau-Cter) and NSE as loading control; (bottom) Sandwich ELISA; histograms indicate mean ± SEM, *n* = 4 independent experiments, ****P < 0.0001, unpaired *t* test

2H2D11 antibody was generated by immunization with the AcMet11-Tau peptide corresponding to the human Tau sequence from Methionine at position 11 to Glycine at position 21, encoded by exon 1 and shared by all human Tau isoforms. A cysteine residue has been added to the peptide C-terminus for coupling to a Keyhole limpet hemocyanin (KLH). Immunizations and generation of hybridomas were carried out by Agro-Bio (La Ferté Saint-Aubin, France), in accordance with applicable regulations. The peptides were synthesized by standard chemical peptide synthesis and purity was analyzed by HPLC (Agro-Bio, France). Balb/c mice were immunized subcutaneously with 3 boosts at days 14, 45 and 63. The lymphocytes from the spleen of the mouse displaying the highest titer were then fused with NS1 myeloma cells, according to the previously described method [34]. Supernatants from the resulting hybridomas were screened by indirect ELISA against the following peptides: AcMet11-Tau, Met11-Tau and AcMet1-Tau (Fig. 1c); the AcMet1-Tau peptide starts at Methionine 1 of human Tau with an N-α-terminal acetylation. ELISA screenings allowed selection of clones that specifically detect the AcMet11-Tau with a slight or without any cross-reactivity with either Met11-Tau or AcMet1-Tau peptides. Isotype and the type of light chain have been determined for the selected hybridomas (The SBA Clonotyping™ System/HRP kit, # MBS678003). The hybridoma 2H2 was further subcloned and the clone that produces 2H2D11 antibody (IgG2a, Kappa) was selected. The specificity of 2H2D11 towards N-α-terminally acetylated methionine11 of Tau protein was consistently validated by ELISA, western blotting and immunohistochemistry.

An antibody against total Tau proteins was also generated by the same procedure, using Met11-Tau peptide (MEDHAGTYGLG) coupled to KLH for immunization. We selected the clone 7C12 (IgG1, Kappa) that recognizes all the 3 Tau-peptides.

### Hybridoma cell culture for antibody production

Hybridoma cell culture was performed in the 2-compartment CELLine CL 1000 bioreactor flask (90-005, Integra Biosciences, France), using hybridoma serum free medium (12045076, Gibco BRL) supplemented with 2 mM L-glutamine and 50 units/mL penicillin/streptomycin, and maintained in a 5% CO_2_ humidified incubator at 37 °C. High cell density culture was performed over a period of 20–30 days as previously described [35] with minor modifications. Hybridoma cells (2.5 × 10^7^ in 15 mL) were seeded in the cell compartment and the nutrient compartment was filled with 500 mL of the cell culture medium. The cell compartment medium harvest/replenish and the nutrient medium exchange were performed every 3 days. The harvest from cell compartment was centrifuged at 250 × *g* for 5 min and the supernatant was then filtered with a 0.22-${\upmu}$m filter and kept frozen at − 20 °C until use for antibody purification. Cell viability was checked during each cell compartment medium harvest by Trypan blue dye exclusion, using a cell counting chamber (Cat. No. 87144F, Kova International, Garden Grove, CA).

### Purification of monoclonal antibodies using the AKTA® System

Monoclonal antibodies were purified from hybridoma culture supernatants (pool of 5–8 cell compartment medium harvests) using an AKTA® FPLC system (Cytiva, Marlborough, MA). This automated chromatography platform was equipped with a 5-mL HiTrap Protein A or Protein G affinity column (Cat. No. 17‑0403‑01 or 17‑0405‑01, Cytiva, Marlborough, MA). The column was first equilibrated with binding buffer (20 mM sodium phosphate, pH 7.0). Culture supernatant samples were injected onto the column, and after wash with binding buffer, elution was performed using 100 mM glycine–HCl, pH 2.7. Successive elution fractions (approximately 10–15 mL total volume per purification run) were collected and analyzed for antibody presence by SDS-PAGE electrophoresis (NuPAGE Novex Bis–Tris 4%–12% gels; Cat. No. NP0329BOX, Invitrogen). A 6 μL aliquot of each fraction was loaded onto the gel. Following migration, gels were stained with Coomassie Brilliant Blue G-250 (0.1% Coomassie in 50% ethanol and 10% acetic acid) and subsequently de-stained in a solution containing 7% acetic acid and 10% ethanol. This analysis allowed identification of elution fractions containing the purified antibody. Positive fractions were pooled and dialyzed against PBS using a 14 kDa dialysis tubing cellulose membrane (D9777-100FT, Sigma Aldrich), overnight at 4 °C under agitation. The antibody solution was then quantified using the BCA assay kit (Cat. No. 23225, Pierce, Rockford, IL). If the final antibody concentration was below 1 mg/mL, a concentration step was performed using Amicon Ultra-15 centrifugal filter units with a 30 kDa molecular weight cut-off (Cat. No. UFC903008, Merck Millipore, Burlington, MA). Two quality control steps were performed: (1) Integrity assessment by SDS-PAGE analysis of antibody samples treated or untreated with the reducing agent DTT (dithiothreitol), to confirm that the purification procedure did not induce antibody denaturation, and (2) Functionality verification by Western blot, using protein lysates from cells expressing or not the target antigen. This confirmed the antigen-binding functionality of the purified antibody. If both quality controls were satisfactory, the antibody was considered suitable for downstream applications. For immunotherapy-related application, endotoxin levels were measured using the Pierce™ chromogenic endotoxin Quant Kit (Thermo Fisher Scientific). Endotoxin concentrations were found to be 0.66 EU/mL for the purified 2H2D11 antibody and 3.14 EU/mL for the control IgG. An endotoxin level of less than 10 EU/mL is recommended for preclinical studies involving in vivo antibody administration [36].

### Indirect ELISA

Nunc 96-well microtiter plates (Maxisorp F8; Cat. No. 439454, Nunc, Inc., Rochester, NY) were coated overnight at 4 °C with 100 ng/well of AcMet11-Tau peptide, Met11-Tau peptide or AcMet1-Tau peptide in 50 mM NaHCO_3_, pH 9.6. After 3 washes with PBS containing 0.05% Tween (PBS-T), plates were blocked with 0.1% casein solution (PBS) at 37 °C for 1 h, followed by incubation with hybridoma supernatants, or purified 2H2D11 or 7C12 antibody (dilution in PBS containing 0.2% BSA), or mouse plasma (dilution 1:600 in PBS with 0.2% BSA) for 2 h at room temperature. After 3 washes with PBS-T, immunodetection was performed by using a goat anti-mouse IgG-horseradish peroxidase (HRP) antibody (A3673; Sigma) at 1:4000 dilution in PBS-BSA 0.2%, at 37 °C for 1 h. After 5 washes with PBS-T, detection was performed using Tetramethyl benzidine substrate (T3405, Sigma) for 30 min at room temperature. The assay was stopped with H_2_SO_4_ and absorbance was read at 450 nm with a Multiskan Ascent spectrophotometer (Thermo Labsystems, Vantaa, Uusimaa, Finland). All the peptides were synthesized by standard chemical peptide synthesis and purity was analyzed by HPLC (ProteoGenix, Schiltigheim, France).

### Sandwich ELISA

Nunc 96-well plates (VWR International, Radnor, PA) were coated with 100 µL (100 ng) of 2H2D11 antibody (for detection of AcMet11-Tau) or 7C12 antibody (detection of total Tau) in Carbonate buffer (NaHCO_3_ 0.1 M, Na_2_CO_3_ 0.1 M, pH 9.6) overnight at 4 °C. The plates were subsequently blocked with a WASH 1 × buffer (INNOTEST hTau Ag kit, FUJIREBIO, Tokyo, Japan) containing 0.1% casein at 37 °C for 1 h and washed with WASH 1 × buffer 3 times. Protein samples were standardized at 1 mg/mL and diluted in SAMPL DIL buffer (INNOTEST hTau Ag kit, FUJIREBIO). Protein samples and biotinylated antibodies (HT7/BT2, INNOTEST hTau Ag kit, FUJIREBIO) were added and the plates were incubated at room temperature overnight. The wells were washed four times, then incubated with Peroxidase-labeled streptavidin at room temperature for 30 min and washed four times. Detection was performed using the Tetramethyl benzidine substrate for 30 min at room temperature. The assay was stopped with H_2_SO_4_ and absorbance was read with the Multiskan Ascent spetrophotometer (Thermo Labsystems) at 450 nm.

Tau phosphorylated at Ser396 was detected by ELISA using the Human Tau [pS396] ELISA Kit (Cat. No. KHB0041, Invitrogen) according to manufacturer’s instructions.

### Stereotaxic injections

One-month-old heterozygous Thy-Tau30 transgenic mice were anesthetized with IP injection of ketamine (100 mg/kg) and xylazine (20 mg/kg) mix. The animals were positioned on a stereotactic device (David Kopf Instrument) and equivalent amounts of Lvs (445 ng of p24) or PBS (2.5 μl) were injected bilaterally into the CA1 region of the hippocampus, at the following coordinates with respect to the bregma: antero-posterior (AP), − 2.5 mm; medial–lateral (ML), − 1 mm (right side) and + 1 (left side); and dorso-ventral (DV), − 1.8 mm. Injection was made at a rate of 0.25 μL/min, using a 10 μL glass syringe with a fixed needle (Hamilton; Dutscher, Brumath, France). Two months post-injections, mice were deeply anesthetized with pentobarbital sodium (50 mg/kg, IP), then transcardially perfused, first with cold NaCl (0.9%) and then with 4% paraformaldehyde in 0.1 mol/L PBS (pH 7.4) for 20 min. Brains were post-fixed for 1 day in 4% paraformaldehyde, then placed in 20% sucrose for 24 h, frozen 1 min in Isopentane at − 40 °C, and then kept at − 80 °C until use.

The same procedure was used for intracerebral injection of monoclonal antibodies. Thy-Tau30 mice (3.5-month-old) received bilateral stereotaxic injections of 2 μg (1 μl) of 2H2D11 or control IgG (see below) in the hippocampus (coordinates relative to Bregma: AP: − 2.5, ML: ± 1, DV: − 2.3). Five days post-injection, brains were processed as described above.

### Passive immunotherapy

Repeated IP injections of 2H2D11 antibody (IgG2a isotype) or control IgG (IgG2a isotype control, purified from B69 hybridoma (ATCC® HB-9437™) were performed in heterozygous Thy-Tau22 at the dose of 10 mg/kg (Fig. S1). In the same experimental procedure, littermate WT mice were injected with either PBS or control IgG. Mice received their first IP injection at 3 months of age and then every 2 weeks until behavioral evaluation at 7 months of age. Blood samples were collected from tail vein and plasma was recovered by centrifugation and kept at − 20 °C until use in ELISA assays. Mice received a last injection one week before sacrifice (at 8 months of age). The animals were sacrificed by cervical dislocation and brains removed. The right hemispheres were post-fixed for 7 days in 4% paraformaldehyde, then placed in 20% sucrose for 24 h and kept frozen at − 80 °C until use. The left hemispheres were used to dissect out hippocampus by using a coronal acrylic slicer (Delta Microscopies, Mauressac, France) at 4 °C and stored at − 80 °C for biochemical analyses.

### Immunohistochemistry

Mouse serial free-floating brain coronal sections (40 μm) were obtained using a cryostat (Leica Microsystems GmbH, Germany) and kept in a PBS-azide (0.2%) at 4 °C until use. Human post-mortem hippocampal samples were fixed after autopsy and processed into free-floating cryosections following the same fixation, sectioning, and immunohistochemical procedures as those used for mouse brain sections. Brain sections were washed with PBS-Triton (0.2%), treated for 30 min with 0.3% H_2_O_2_ and nonspecific binding was blocked with MOM (Mouse on Mouse) blocking reagent (Vector Laboratories, Burlingame, CA) or horse serum (1:100 in PBS; Vector Laboratories) for 1 h. The sections were then incubated with the primary antibody (Table S2) in PBS-Triton 0.2% overnight at 4°C. After 3 × 10 min washes, labeling was amplified using a biotinylated anti-mouse or anti-rabbit IgG (1:400 in PBS-Triton 0.2%; Vector Laboratories) for 1 h, followed by the ABC kit (1:400 in PBS; Vector Laboratories), and labeling was completed using 0.5 mg/mL DAB (Vector Laboratories) in 50 mmol/l Tris–HCl, pH 7.6, containing 0.075% H_2_O_2_. Brain sections were mounted on SuperFrost slides, dehydrated through a graded series of alcohol and toluene, and then mounted with Vectamount medium (#H5000; Vector Laboratories) and coverslips (#LCO2460M; Labelians) for microscopic analysis. Tissue sections were digitized using an automated slide scanner (Zeiss AXIOSCAN Z1, Carl Zeiss, Oberkochen, Baden‑Württemberg, Germany) coupled with a high-resolution microscope (× 20 objective). The resulting virtual images were initially saved in CZI format and then converted to TIFF format using ZEN imaging software for export and further analysis. Quantification of immunostaining was performed using ImageJ software. Depending on the analysis type, either the number of immunopositive cells or the percentage of labeled surface area was quantified. Briefly, images were converted to 8-bit grayscale, and a thresholding procedure was applied to isolate the signal of antibody staining. This allowed for the measurement of either the number of positive events (cells) or the percentage of the area stained. For each section, the total surface area of the hippocampus was also measured and recorded.

### Immunofluorescence

Mouse brain sections were washed with PBS-Triton 0.2%, and nonspecific binding was blocked through incubation with MOM (1:100 in PBS-Triton 0.2%; Vector Laboratories) for 1 h. For co-labeling, the sections were incubated with 2H2D11 antibody (Table S2) in PBS-Triton 0.2% overnight at 4°C. After 3 washes (10 min), the sections were incubated with either Tau p-Ser199 or Tau p-Ser422 antibody (Table S2) in PBS-Triton 0.2% overnight at 4°C. Sections were then washed and incubated with goat anti‑rabbit IgG conjugated to Alexa Fluor 488 (Cat. No. A‑11008, Invitrogen) and anti-mouse IgG conjugated to Alexa Fluor 568 (Cat. No. A-11004, Invitrogen), 1:1000 in PBS for 1 h at room temperature. After 3 × 10 min washes, the sections were mounted with Vectashield containing DAPI (Vector Laboratories) to label the nuclei. Confocal microscopy was performed on a Zeiss LSM 710 inverted confocal microscope (× 63 oil objective). Images were collected in the z direction at 1 ${\upmu}$ m or 0.8 ${\upmu}$m intervals. For quantification of double-labeled neurons, maximum intensity projection images from double-immunolabeled sections were analyzed using a custom ImageJ macro. Neurons exhibiting green or red immunofluorescence were first identified based on fluorescence intensity thresholds. Double-positive cells were then determined, allowing estimation of the proportion of double-positive cells. This analysis was used to support the qualitative assessment of the preferential association of AcMet11-Tau with pathological Tau-positive neurons.

### Peptide blocking experiments

Before proceeding to Western blot or immunohistochemistry, the 2H2D11 antibody was incubated with agitation overnight at 4 °C, in blocking buffer without or with excess of the blocking peptide (molar ratio: 1:50). Thereafter, the antibody samples were used to perform staining protocol as described in Western blotting and immunohistochemistry sections.

### Protein extraction

Cells were washed with PBS and collected in ice-cold RIPA buffer (150 mM NaCl, 1% NP40, 0.5% sodium deoxycholate, 50 mM Tris–HCl, pH 8.0) containing a protease inhibitor cocktail (Complet mini EDTA-Free tablets; Cat. No. 11836170001, Roche, Basel, Switzerland). After sonication (20 pulses at 40 Hz) and homogenization for 30 min at 4 °C under agitation, the supernatants were recovered after centrifugation at 12,000 × *g* for 10 min at 4 °C.

Brain tissue samples were processed following a standardized protocol. Approximately 100 mg of human brain tissue were homogenized by sonication (30 pulses at 40 Hz) in 1 mL of cryopreservation buffer composed of 10 mM Tris–HCl and 0.32 M sucrose (pH 7.4), supplemented with a protease inhibitor cocktail (Complet mini EDTA-Free tablets, Roche). For mice, half of the hippocampus was homogenized in 200 μL of cryopreservation buffer. Homogenates were stored at –80 °C until use. For total protein extraction, an aliquot of the tissue suspension was mixed (1:1, *v*/*v*) with 2 × RIPA ice-cold buffer, and protein extraction was carried out as described above. Protein concentrations were determined using the BCA Assay Kit (Pierce) and the extracts were kept at − 80 °C until use in Western blotting or ELISA.

### Western blotting

For total proteins, extracts were standardized at 1 mg/mL with LDS 2 × supplemented with a reducing agent (Invitrogen) and denatured at 100 °C for 10 min. Proteins were then separated with SDS-PAGE using precast 4%–12% Bis–Tris NuPage Novex gels (Invitrogen). Proteins were transferred to 0.45 μm nitrocellulose membranes (Amersham Hybond ECL, Amersham, Little Chalfont, UK), which were saturated with 5% non-fat milk or bovine serum albumin (Sigma) in TNT buffer (140 mM NaCl, 0.5% Tween20, 15 mM Tris, pH 7.4). Membranes were then incubated with the primary antibody overnight at 4°C, washed with TNT buffer 3 times for 10 min, incubated with the HRP-conjugated secondary antibody (Vector) for 45 min at room temperature and washed again. Immunolabeling was visualized using a chemiluminescence kit (ECL #RPN2106; Amersham) and the LAS-4000 acquisition system (Fujifilm, Tokyo, Japan). The antibodies are listed in Table S2.

### Behavioral tests

To avoid bias, animals were randomly allocated to experimental groups, and behavioral testing was performed by investigators blinded to treatment conditions. Only male mice were used in behavioral testing.

#### Actimetry

Spontaneous locomotor activity was assessed using actimetry arenas (Bioseb, LE8816; 45 × 45 × 35 cm^3^) lightened at 40 lux. Each arena was equipped with two infrared beam frames: one positioned 2 cm above the floor to detect horizontal movements, and the other at 6 cm to assess vertical activity (rearing). Mice were habituated to the testing room for at least 30 min prior to recording. During the test session, each mouse was allowed to freely explore the arena for 10 min. Locomotor parameters including velocity and total distance moved, were recorded using the Actitrack software (Bioseb, Vitrolles, France).

#### Elevated plus maze (EPM)

Anxiety-like behavior was evaluated using the EPM. The maze consisted of four arms (35 cm long × 5 cm wide), arranged in a plus configuration and elevated 60 cm above the floor. Two arms were open (no walls), and two were enclosed by 15 cm-high opaque walls. Mice were placed in the center of the maze and allowed to explore freely for 5 min. Locomotor activity and the time spent in each arm were recorded and analyzed using the EthoVision XT tracking system (Noldus, Wageningen, The Netherlands).

#### Y-maze

Short-term spatial memory was assessed using the Y-maze, composed of three opaque arms (22 cm long × 6.4 cm wide × 15 cm high) arranged at 120° angles. Spatial cues were positioned on the surrounding walls to support spatial orientation. For each animal, the roles of the “start”, “novel”, and “other” arms were randomly assigned. In the learning phase, the novel arm was blocked by an opaque door. The mouse was placed in the start arm and allowed to explore the two accessible arms (start and other) for 5 min. After a 2-min inter-trial interval in the home cage, the novel arm was opened, and the mouse was placed again in the start arm for the test phase, where it could explore all three arms for an additional 5 min (from the time the mouse first left the start arm). Time spent in each arm during both phases was recorded using EthoVision XT (Noldus).

#### Barnes maze

Spatial learning was assessed using the Barnes maze task. The maze consisted of a white circular PVC platform (120-cm diameter), placed on a swivel system in the center of the room, elevated 80 cm above the floor and brightly lit (370 lux). Forty equally spaced holes (5 cm in diameter) were arranged along the perimeter, 5 cm from the edge. Only one hole led to a dark escape box under the platform. Visual cues were placed on the walls surrounding the maze to facilitate spatial orientation. A camera positioned above the maze recorded animal behavior, which was analyzed using EthoVision XT (Noldus). In the habituation phase (day 0), the mice explored the maze for 2 min. If they failed to find the escape hole, they were gently guided toward it and allowed to remain inside for 30 s. The escape hole location was randomized for each mouse. In the acquisition phase (days 1–4), mice underwent four trials per day to learn the escape hole location. At the start of each trial, animals were placed under an opaque cylinder in the center of the platform for 10 s. Upon cylinder removal, mice had 3 min to locate the target hole. Animals that failed to do so on day 1 were guided to the hole and remained in the escape box for 30 s. Between trials, mice were returned to their home cage for a 15-min inter-trial interval. The platform was cleaned with 70% ethanol and rotated by 45° daily to prevent olfactory cue reliance. The order of testing was pseudo-randomized across animals to avoid experimental bias.

For the acquisition phase, the number of primary errors (number of times the mouse explores the other holes before finding the target one), the total errors and the distance moved were recorded.

### RNA extraction and quantitative RT-PCR

Total RNA was extracted from dissected mouse hippocampi using the RNeasy Lipid Tissue Mini Kit (Cat. No. 74804, Qiagen, Hilden, Germany), according to the manufacturer's instructions. RNA concentrations were determined using a NanoDrop ND-1000 spectrophotometer at 260 nm. RNA purity was assessed using A260/A280 and A260/A230 absorbance ratios. Extracted RNA samples were stored at –80 °C until use.

Reverse transcription was performed using 1 µg of total RNA and the High-Capacity cDNA Reverse Transcription Kit (Cat. No. 4368814, Applied Biosystems, Foster City, CA), in a T Gradient Biometra thermocycler, following the thermal conditions: 25 °C for 10 s, 37 °C for 2 h, and 85 °C for 5 s. The resulting cDNAs were stored at –80 °C until further analysis. Quantitative PCR (qPCR) was carried out on an Applied Biosystems Prism 7900 using a 1:20 dilution of cDNA and the Power SYBR™ Green PCR Master Mix (Cat. No. 4367659, Applied Biosystems), or 1:10 dilution of cDNA and TaqMan gene expression master mix (Cat. No. 4369016, Life Technologies, Carlsbad, CA). The primers (Table S3) were used at a final concentration of 0.1 nM. The qPCR program was as follows: 2 min at 50 °C, 10 min at 95 °C, followed by 40 cycles of 15 s at 95 °C and 25 s at 60 °C. A final step included 15 s at 95 °C, followed by 1 min at 60 °C, and a gradual temperature increase to 95 °C.

*PPIA* (peptidyl-prolyl isomerase A), a ubiquitously expressed housekeeping gene with stable expression under experimental conditions, was used as the endogenous reference gene for normalization. All reactions were performed in triplicates, and relative gene expression was calculated using the comparative Ct method (ΔΔCt).

### Statistical analysis

Statistical analyses were performed using Prism 10 software (GraphPad Software Inc., San Diego, CA). Data are presented as mean ± standard error of the mean (SEM). The normality of data distribution within groups was assessed using the Shapiro–Wilk test. Depending on the number of experimental groups, comparisons were performed using either unpaired *t*-test or one-way ANOVA followed by appropriate multiple comparison post hoc tests (Tukey's HSD or Fisher’s LSD). Correlation analyses between continuous variables were carried out using Spearman’s rank correlation. For all statistical tests, a *P*-value < 0.05 was considered statistically significant. The specific details are indicated in the figure legends.

## Results

### Identification of AcMet11-Tau and generation of a specific antibody

By combining immunoprecipitation, primary amine labeling using a covalently-linked biotin prior to trypsic digestion and capillary LC–MS/MS, we previously identified 18 amino-truncated Tau species in human brain samples [11]. Repeating this experiment, we identified in occipital brain sample a Tau variant starting at Met11 residue, bearing an N-terminal acetylation (hereafter referred to as AcMet11-Tau) (Fig. 1a). To validate this finding and to investigate the pathophysiological relevance of AcMet11-Tau, we developed a monoclonal antibody specifically targeting this variant, termed 2H2D11 (Fig. 1b). Indirect ELISA using different Tau peptides showed 2H2D11 to be specific for the AcMet11-Tau peptide (Fig. 1c). Notably, this antibody did not recognize either the unmodified Met11 (Met11-Tau peptide) or the non-truncated Tau peptide containing N-α-acetyl-Methionine in a different Tau sequence context (AcMet1-Tau peptide), thereby confirming the high sequence- and modification-specificity of the 2H2D11 antibody. To further validate its specificity in a cellular context, we generated stable SH-SY5Y neuroblastoma cell lines by using tetracycline-inducible expression vectors containing the coding sequence of either full-length (FL-Tau) or Met11-Tau. Western blot analysis revealed that 2H2D11 detected Tau expression exclusively in Met11-Tau-expressing cells (hereafter referred to as AcMet11-Tau cells), with no signal in FL-Tau cells (Fig. 1d). Specificity was further confirmed by peptide blocking experiments, where pre-incubation of 2H2D11 with the AcMet11-Tau peptide abolished the signal. Additionally, a sandwich ELISA using 2H2D11 demonstrated strong immunoreactivity only in extracts from AcMet11-Tau-expressing cells, while FL-Tau cell extracts yielded signals comparable to blank controls (Fig. 1d, bottom). Collectively, these results establish 2H2D11 antibody as a highly specific and reliable tool for the detection of the AcMet11-Tau variant, providing a valuable resource for further investigation of its pathophysiological role.

### AcMet11-Tau is an early pathological marker in a mouse model of Tau pathology

**Fig. 2:**
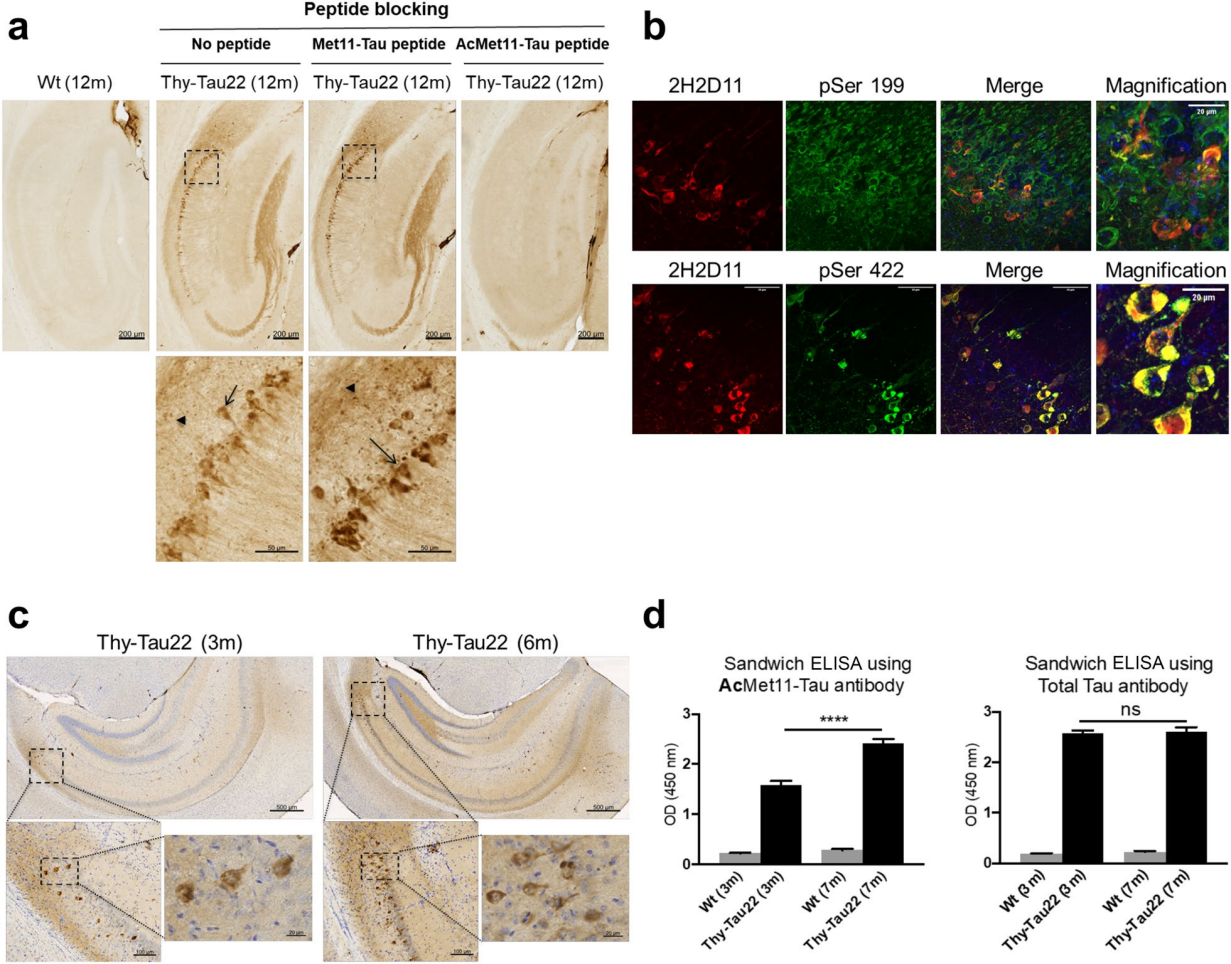
AcMet11-Tau is an early pathological species in Thy-Tau22 mice. **a** Representative immunohistochemical analysis of coronal brain sections, using 2H2D11 antibody that labels neurons bearing neurofibrillary tangles (arrows) and neuritic processes (arrowheads) in the hippocampus of 12-month-old Thy-Tau22 compared to littermate WT; blocking experiments with Tau-peptides indicate the specificity of AcMet11-Tau immunostaining. Scale bars = 200 µm and 50 µm (inset magnification, bottom). **b** Representative confocal images of double immunostaining using 2H2D11 antibody with either pSer199 (Top) or pSer422 (bottom) in the hippocampal CA1 region of 9-month-old Thy-Tau22 mice. Scale bars = 50 µm and 20 µm (merge magnification). Analysis of co-labeled neurons indicates that 2H2D11 is present in a subset of pSer199-positive neurons (17/51) and in most pSer422-positive neurons (17/18), supporting its preferential association with neurons exhibiting pathological Tau. **c** Immunohistochemical labelling with 2H2D11 of coronal brain sections from 3- and 6-month-old Thy-Tau22 mice. Scale bars = 500 µm (top), 100 µm and 20 µm (bottom). **d** 2H2D11 antibody-based ELISA using hippocampal proteins of Thy-Tau22 and their littermate WT mice of 3 and 7 months of age. Histograms indicate mean ± SEM (*n* = 5/group). ****P < 0.0001, one-way ANOVA followed by a post hoc Fisher’s LSD test

To investigate a potential association between AcMet11-Tau and NFD, we analyzed brain sections from 12-month-old Thy-Tau22 mice, a model that progressively develops hippocampal tau pathology from 3 months of age and exhibits memory impairments by 7 months [24, 25]. Immunohistochemical staining with the 2H2D11 antibody revealed no detectable signal in WT littermate mice, whereas Thy-Tau22 mice showed robust hippocampal labelling. Notably, 2H2D11 specifically localized to NFT-like inclusions and neuritic processes (Fig. 2a).

To confirm signal specificity, we performed peptide-blocking assays. Pre-incubation of 2H2D11 with the AcMet11-Tau peptide abolished immunoreactivity, whereas pre-incubation with the non-α-acetylated Met11-Tau peptide had no effect (Fig. 2a). These findings support the α-acetylation- and sequence-specific recognition of AcMet11-Tau in situ. Furthermore, we performed double labeling experiments using 2H2D11 and antibodies specific to phosphorylated Tau. The pSer199 antibody broadly labels Tau in neurons whereas the pSer422 antibody specifically detects abnormally phosphorylated pathological Tau in neurons with NFTs. Co-labeling revealed that the 2H2D11 antibody selectively labeled neurons that were positive for pSer422, but not all pSer199-positive neurons (Fig. 2b), indicating that AcMet11-Tau is preferentially associated with neurons undergoing NFD. Strikingly, AcMet11-Tau was detected during early stages of disease progression, largely ahead of the onset of memory deficits. Indeed, immunohistochemical analyses of hippocampal brain sections (Fig. 2c) as well as sandwich ELISA using hippocampal protein extracts (Fig. 2d) demonstrated the presence of AcMet11-Tau as early as 3 months of age in Thy-Tau22 mice. These findings suggest that AcMet11-Tau emerges early in the pathological cascade and may contribute to or serve as an early biomarker of Tau pathology.

### AcMet11-Tau is a pathological species specifically detected in the AD brain areas along pathology progression

**Fig. 3:**
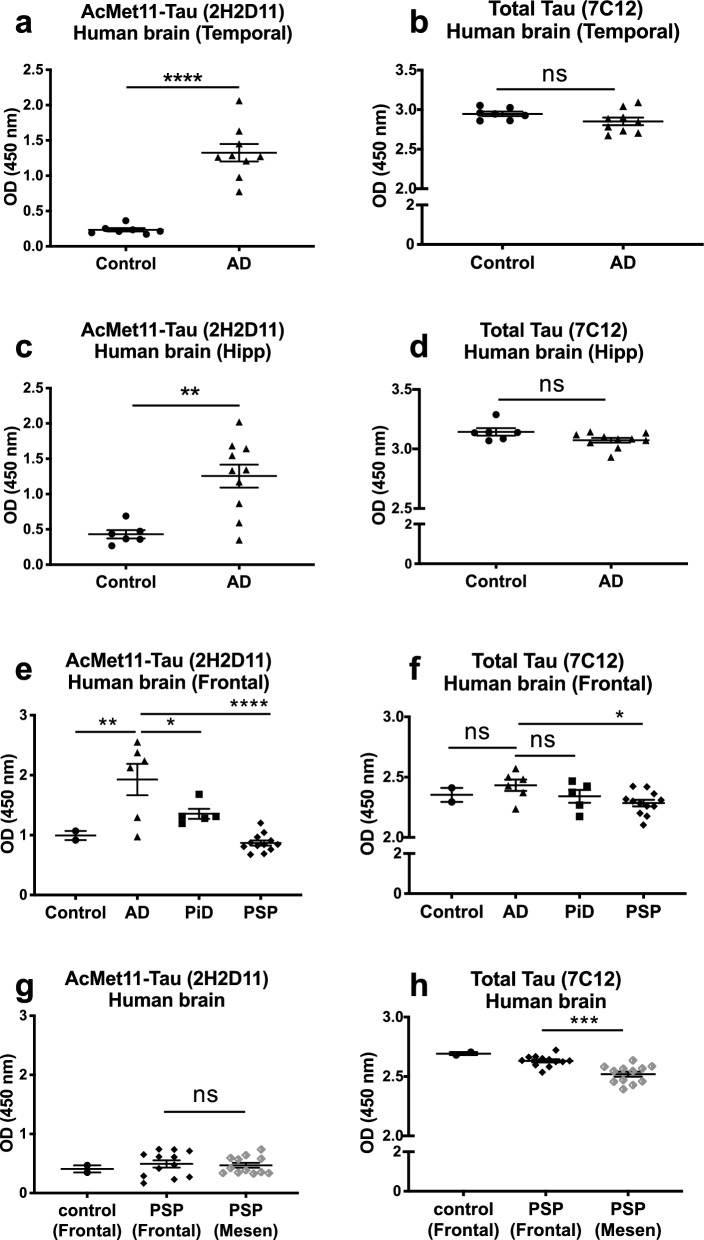
AcMet11-Tau is distinctively detected in AD brains. **a**–**d** Protein extracts from temporal and hippocampal regions of controls (*n* = 7 and 6) and AD cases (*n* = 9 and 10) were used in 2H2D11 and 7C12 antibody-based sandwich ELISA (mean ± SEM, **P = 0.0022, ****P < 0.0001, unpaired *t* test). **e**, **f** Protein extracts from frontal region of controls (*n* = 2), AD (*n* = 6), PiD (*n* = 5) and PSP cases (*n* = 12) were used in 2H2D11 and 7C12 antibody-based sandwich ELISA (mean ± SEM, **P = 0.0029 (AD vs. control), *P = 0.0113 (AD vs. PiD), ****P < 0.0001 (2H2D11, AD vs. PSP), *P = 0.0104 (7C12, AD vs. PSP), one-way ANOVA followed by Fisher’s LSD post hoc test). **g**, **h** Protein extracts from mesencephalon and frontal regions of PSP cases (*n* = 12) were used in 2H2D11 and 7C12 antibody-based sandwich ELISA (mean ± SEM, ***P = 0.0001, one-way ANOVA followed by Fisher’s LSD post hoc test)

To assess the presence of AcMet11-Tau in human brain tissues from patients with AD, we first examined hippocampal sections from post-mortem human brains by immunohistochemistry. Using the 2H2D11 antibody, no immunoreactivity was detected in the hippocampal sections from elderly control subjects. In contrast, hippocampal sections from AD patients showed a clear 2H2D11 labeling pattern consistent with NFD, including neuronal tau tangles and dystrophic neurites (Fig. S2). To further characterize AcMet11-Tau, we analyzed protein extracts from the temporal cortex and hippocampus of control subjects and AD cases (Braak stages IV–VI), using a 2H2D11-based sandwich ELISA assay. AcMet11-Tau was significantly detected in AD samples as compared to matched controls (Fig. 3a, c), while total Tau was similarly detected between the two groups (Fig. 3b, d). To examine disease specificity, across tauopathies, we further analyzed frontal cortex of patients with AD along with patients presenting with PiD and PSP, two primary tauopathies. 2H2D11 immunoreactivity clearly discriminated AD cases from the two other tauopathies (Fig. 3e, f). Since Tau pathology in PSP patients was more predominant in the mesencephalon than in the frontal cortex (Fig. S3), we further analyzed AcMet11-Tau expression across two PSP brain regions. AcMet11-Tau remained undetectable, even in the region with robust Tau pathology (Fig. 3g, h), indicating that AcMet11-Tau is not a feature of PSP.

**Fig. 4:**
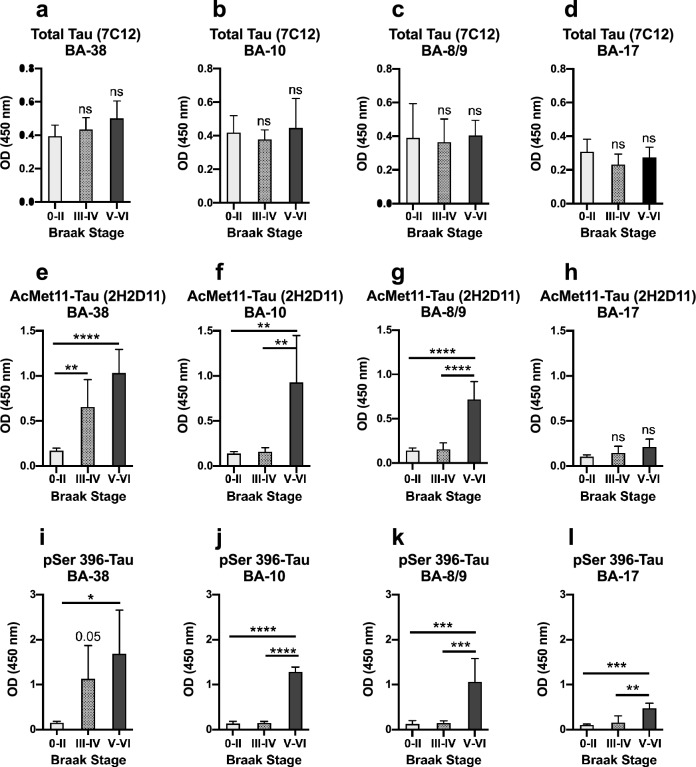
AcMet11-Tau exhibits regional association with Tau pathology throughout AD progression. **a**–**l** ELISA analysis of tissues from different brain areas and individuals at various stages of NFD progression. **a**–**d** 7C12 antibody (total Tau) was used as a control for Tau protein detection within the samples. **e–h** AcMet11-Tau form was detected by 2H2D11 antibody. **i-l** Phosphorylated Tau was detected using the pSer396 antibody. Data are expressed as the mean optical density ± SD (*n* = 6 (stages 0-II), *n* = 5 (stages III-IV), *n* = 3 (stages V-VI)). *P < 0.05, **P < 0.01, ***P < 0.001, ****P < 0.0001, one-way ANOVA analysis followed by a Tukey HSD post-hoc test

Moreover, we investigated whether AcMet11-Tau is an early pathological marker in AD. To do this, we conducted ELISA-based assay in samples from different brain Brodmann areas (BA) of individuals spanning Braak stages 0 to VI. Prior to analysis, all brain samples were biochemically characterized by immunoblotting to confirm the typical Tau pathology profile, allowing the validation of Braak stages (Fig. S4a). In BA-38 (anterior temporal pole, affected from Braak stage III), while no significant differences were observed in the total Tau levels detected by the 7C12 antibody across the different groups (Fig. 4a), analysis revealed a significant detection of AcMet11-Tau immunoreactivity in Braak stage III–IV and V–VI groups as compared to the Braak stage 0–II group (Fig. 4e). In BA-10 (frontal pole, affected from Braak stage V) and BA-8/9 (dorsolateral prefrontal cortex, affected in later stages), AcMet11-Tau was significantly detected only in the Braak stage V-VI group (Fig. 4f). As expected, AcMet11-Tau detection in BA-17 (primary visual cortex, relatively spared even at the late stages of AD) was not significant (Fig. 4h). A similar detection pattern, consistent with Braak stages and brain regions, was observed using a reference ELISA analysis targeting pathological hyperphosphorylated Tau at pSer396 (Fig. 4i–l). In line with this observation, ELISA optical density values obtained with AcMet11-Tau antibody were positively associated with those measured using pSer396 antibody (Fig. S4b). Collectively, these findings indicate that the AcMet11-Tau protein represents an AD disease-specific Tau species, closely linked to the pathological Braak staging. Its enrichment in vulnerable cortical regions in AD underscores its potential diagnostic and biomarker utility in AD.

### AcMet11-Tau potentiates Tau pathology development in a transgenic mouse model of Tau pathology

**Fig. 5:**
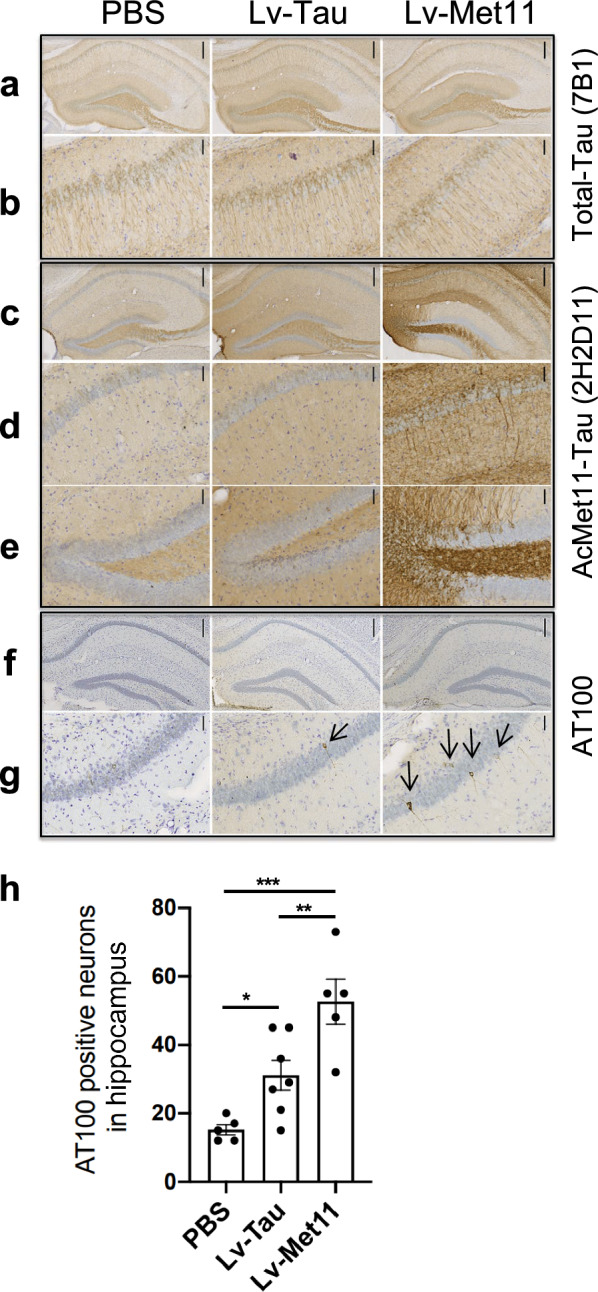
AcMet11-Tau expression in transgenic mice induced an increase in the number of neurofibrillary tangle-bearing neurons. Thy-Tau30 transgenic mice were bilaterally injected with lentival vectors (Lv), into CA1 region of the hippocampus (bregma −2.54) and analyzed 2 months later. **a–g** Immunohistochemical analysis of coronal brain sections using: (**a**, **b)** Total-Tau antibody (7B1); (**c–e)** 2H2D11 antibody; and (**f**, **g)** AT100 that labels neurons bearing NFTs (arrows). Panels **b**, **d** and **g** are higher-magnification images of CA1 region; panel **e** shows higher magnification of dentate gyrus. Scale bars = 200 µm (**a**, **c**, **f**), and 50 µm (**b**, **d**, **e**, **g**). **h** Graph represents the number of AT100-positive neurons quantified in 3 sections from left or right hemisphere (bregma: −1.7 or −1.9, −2.46 or −2.54 and −3.16 or −3.4). Data are expressed as mean ± SEM (5–7 hemispheres/group). *P = 0.0274, **P = 0.0005, ***P = 0.0001, one-way ANOVA followed by Fisher’s LSD post hoc test

To evaluate the AcMet11-Tau pathogenicity, we compared the impact of its overexpression versus FL-Tau in the brains of Tau transgenic mice using Lv designed for neuron-specific expression [30]. We performed stereotaxic injections of Lv into the hippocampus of one-month-old Tau transgenic mice, a developmental stage at which Tau pathology is absent [27]. Prior to stereotaxic injections, we confirmed that both Lv batches (Lv-Tau and Lv-Met11) drove comparable levels of expression in primary neuronal cells, and that Met11-Tau was expressed as N-α-acetylated form (Fig. S5). Two months post injections, immunohistochemical analysis of brain sections using an anti-total Tau antibody confirmed similar expression across both experimental groups (Fig. 5a, b). As anticipated, AcMet11-Tau immunoreactivity was weak in transgenic animals injected with PBS, taken as control, or Lv driving the expression of FL-Tau (Lv-Tau), but was robustly detected in the hippocampi of mice receiving Lv-Met11 (Fig. 5c–e). Notably, the accumulation of aggregated and fibrillary Tau forms, assessed using AT100 immunostaining, was modestly elevated in mice injected with the Lv-Tau, compared to PBS-treated controls. In stark contrast, mice overexpressing the AcMet11-Tau variant (Lv-Met11) exhibited a pronounced accumulation of fibrillary Tau aggregates (Fig. 5f–h). These findings strongly suggest that the AcMet11-Tau variant is not merely a passive marker of Tau pathology, but represents a disease-associated Tau species that actively contributes to the acceleration of NFD in vivo.

### Beneficial effects of passive immunotherapy against AcMet11-Tau

**Fig. 6:**
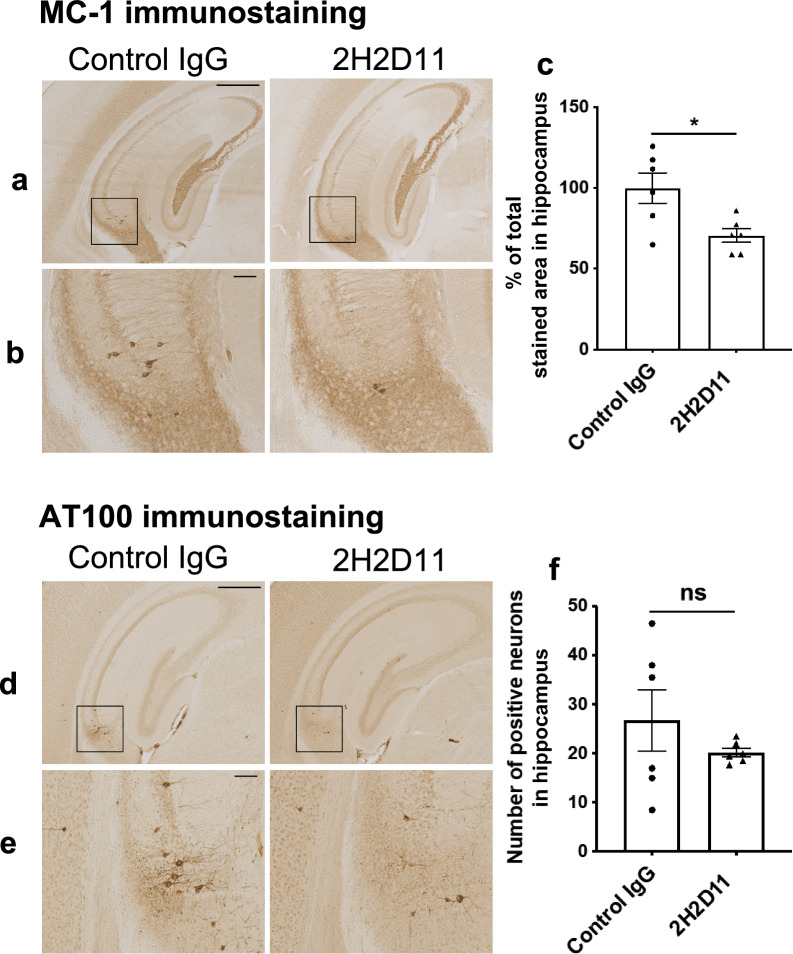
Intracerebral 2H2D11 injection reduces Tau pathology in the hippocampus of transgenic mice. Thy-Tau30 mice (3.5-month-old) received bilateral stereotaxic injections of 2H2D11 or control IgG into the hippocampus. Five days post-injection, brains were processed for immunohistochemistry, using antibodies against pathological Tau protein forms: MC1 and AT100. **a**, **d** Representative images of MC1 and AT100 immunostaining, at bregma − 2.15 mm, scale bar = 500 µm. **b**, **e** Inset magnification, scale bar = 100 µm. **c**, **f** MC1-immunostaining, expressed as a percentage of the total hippocampal area and AT100-immunostaing, expressed as the number of positive neurons; quantifications were performed on three coronal sections per animal spanning bregma − 1.43 to − 3.39 mm; Data are presented as mean ± SEM (*n* = 6 mice per group) and were analyzed using unpaired *t*-test; 2H2D11 injection significantly reduced MC1-labeled area (*P = 0.0169) and showed a trend toward reduced number of AT100-positive neurons (*P* = 0.3211)

To investigate whether targeting AcMet11-Tau could confer protective effects against Tau pathology and associated memory deficits, we evaluated a passive immunotherapy approach using the 2H2D11 antibody. Like a previously described approach [37], we first evaluated the effect of hippocampal injection of 2H2D11 in Tau transgenic mice. Five days following injection, brain sections were analyzed by immunohistochemistry using AT100, an antibody that recognizes aggregated pathological form of Tau, and MC1, a conformational antibody specific to Tau species that precede Tau aggregation and NFD. Mice treated with 2H2D11 exhibited a significant reduction of MC1 immunoreactivity as compared to animals injected with a control IgG (Fig. 6a–c). AT100-positive neurons also appeared to be reduced, albeit not reaching statistical significance within the short treatment window used (Fig. 6d–f). Given that MC1 detects an earlier stage of Tau aggregation process than AT100 [38, 39], our results suggest that immunoneutralization of AcMet11-Tau preferentially impacts early pathogenic Tau species, possibly interfering with the oligomerization process before fibrillary inclusions form. The selective reduction of MC1 staining within just five days highlights the presumable potential of AcMet11-Tau-directed immunotherapy to target upstream, intermediates in the Tau aggregation cascade.

**Fig. 7:**
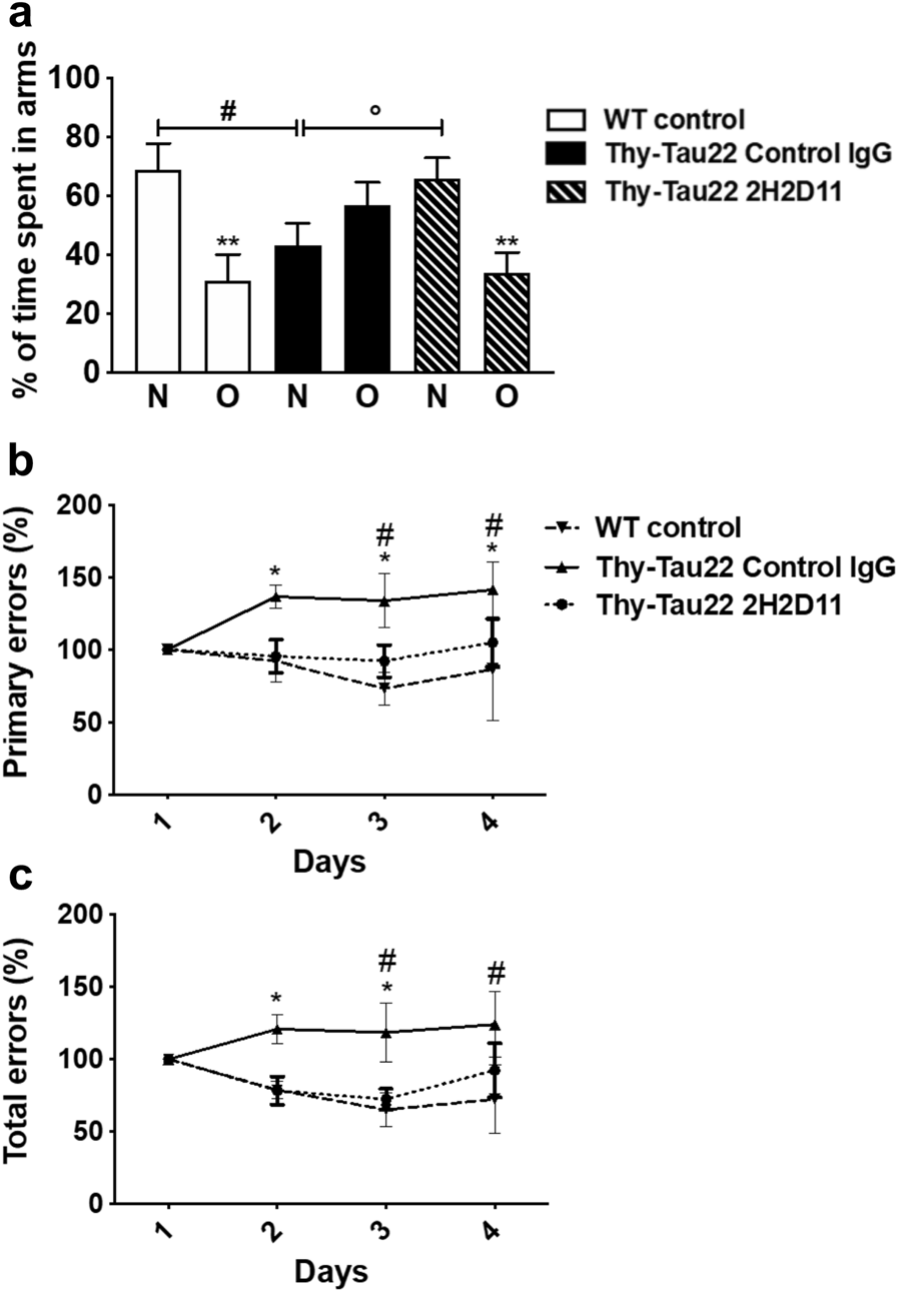
Passive immunotherapy against AcMet11-Tau improves spatial memory and learning of Thy-Tau22 mice. **a** In the Y-Maze task, WT control animals exhibited a preference for the novel (N) over the other (O) familiar arm (**P < 0.01). Thy-Tau22 treated with control IgG showed an absence of preference for N versus O (*P* = 0.21) and the percentage of time spent in the N arm significantly reduced as compared to WT (^#^*P* < 0.05). Conversely, Thy-Tau22 treated with the 2H2D11 antibody exhibited a significant preference for N versus O (**P < 0.01) together with a percentage of time spent in the N arm significantly enhanced as compared to Thy-Tau22 treated with control IgG (°*P* < 0.05). Data were analyzed using one-way ANOVA followed by LSD Fisher test. **b**, **c** In the Barnes maze the number of primary and total errors are as expected significantly different between the WT mice and Thy-Tau22 mice treated with control IgG (*P < 0.05). Thy-Tau22 mice treated with 2H2D11 antibody made significantly less errors in reaching the target than the Thy-Tau22 mice treated with control IgG (^#^*P* < 0.05). Data were analyzed using two-away ANOVA followed by a post-hoc HSD Tukey test. All data are presented as mean ± SEM (*n* = 10–17). Only male mice were used in behavioral testing

**Fig. 8:**
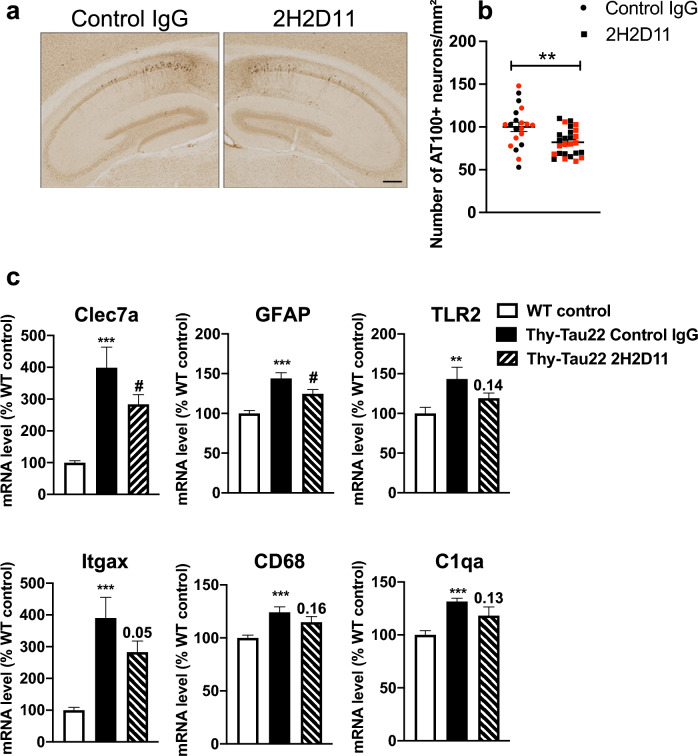
Passive immunotherapy against AcMet11-Tau reduces Tau pathology and modulates selected neuroinflammatory markers in Thy-Tau22 mice. **a** Representative images of AT100 immunostaining at bregma − 2.45 mm, scale bar = 200 µm. **b** AT100-immunostaing, expressed as the number of positive neurons per mm^2^. Quantifications were performed on three coronal sections per animal spanning bregma − 1.43 mm to − 3.39 mm; Data are presented as mean ± SEM (*n* = 20–26 mice per group; females are shown in red) and were analyzed using unpaired *t*-test; **P < 0.01). **c** Treatment with 2H2D11 antibody selectively reduced the mRNA expression of Clec7a, GFAP and Itgax in the hippocampus of Thy-Tau22 mice treated with 2H2D11. Note that the effect on Itgax was modest, with a *P*-value at the threshold of statistical significance (*P* = 0.05). Thy-Tau22 Control IgG versus WT, ***P < 0.001, **P < 0.01; Thy-Tau22 Control IgG versus Thy-Tau22 2H2D11, ^#^*P* < 0.05; one-way ANOVA followed by a post hoc Fisher’s LSD test (*n* = 6/group)

To further assess the therapeutic potential of AcMet11-Tau targeting, we then employed a repeated IP immunization approach using the 2H2D11 antibody or the control IgG (Fig. S1). The Thy-Tau22 mice were injected IP every 15 days, from 3 months (i.e., at pathological onset) to 7 months of age when Tau pathology is ongoing and memory impairments are present but not maximal in this model. As additional controls, we used littermate WT mice with IP injection of control IgG or PBS, to provide a baseline in behavioral evaluations. Only male mice were used in behavioral testing. Antibody titers were measured in plasma by ELISA 120 days after initiation of treatment. The 2H2D11 antibody was readily detected at this time point (Fig. S6a), indicating sustained systemic exposure in treated mice. Longitudinal body weight monitoring showed no effect of immunization with 2H2D11 as compared to control IgG (Fig. S6b, c). Similarly, immunization with 2H2D11 had no impact on animal’s spontaneous motor activity and anxiety-related behavior (Fig. S7a–e). To evaluate the effect of 2H2D11 immunotherapy on memory, we assessed spatial short-term memory using the Y-maze task (Fig. 7a). As expected [24, 40], the Thy-Tau22 mice injected with control IgG displayed impairment of short-term spatial memory, reflected by a lack of preference for the novel arm over the other familiar arm, and a significantly reduced percentage of time spent in the new arm as compared with WT mice. Remarkably, the 2H2D11-injected transgenic mice performed similarly to WT mice, showing a significantly increased percentage of time spent in the new arm as compared to the Thy-Tau22 mice solely treated with the control IgG, supporting restored spatial memory abilities. To further assess cognitive performance, animals were subsequently assessed in the Barnes maze. The general assessment of spatial learning revealed that all animal groups learned the task by locating the target box over the four days of acquisition (Fig. S7f). Although no differences were observed in the path length (Fig. S7f), the Thy-Tau22 mice injected with control IgG made significantly more primary and total errors as compared to the WT littermate controls (Fig. 7b, c). Notably, the Thy-Tau22 mice treated with the 2H2D11 antibody made significantly less errors in reaching the target hole than the Thy-Tau22 mice treated with control IgG, further supporting a beneficial effect of AcMet11-Tau immuno-targeting on spatial learning. In agreement with these behavioral observations, immunohistochemistry staining of the conformational and pathological Tau AT100 epitope showed a statistically significant, albeit moderate, decrease of Tau pathology in the hippocampus of Thy-Tau22 mice injected with the 2H2D11 antibody (Fig. 8a, b). We also assessed some neuroinflammatory markers known to be associated with Tau pathology development in Thy-Tau22 mouse model [26, 41]. As expected, our data indicated that these markers increased significantly in the hippocampus of transgenic Tau mice as compared to the littermate WT controls (Fig. 8c). Notably, the Thy-Tau22 mice treated with the 2H2D11 antibody showed a selective reduction in the expression of Clec7a, GFAP and Itgax compared to the Thy-Tau22 treated with control IgG. While the effects on other markers were limited, these results suggest that passive immunotherapy with 2H2D11 may selectively modulate specific neuroinflammatory pathways associated with Tau pathology.

## Discussion

Neurodegenerative diseases termed tauopathies, including AD, are defined by progressive accumulation of pathological Tau species. While post-translational modifications and truncations of Tau have long been associated with its pathological aggregation, the identity of the specific pathogenic driver forms has remained elusive. Here, we identifed AcMet11-Tau, an N-terminally truncated and N-α-acetylated Tau variant, as a novel pathological species enriched in early degenerating neurons of both transgenic tauopathy models and AD patient brains. Notably, AcMet11-Tau was absent in the two primary tauopathies analyzed, suggesting a selective association with AD. Using a newly generated specific monoclonal antibody, we demonstrated that AcMet11-Tau is not only a marker of NFD, but also a disease-associated Tau species with functional relevance to pathology. Overexpression of AcMet11-Tau in transgenic mice exacerbated Tau pathology, while targeted neutralization via passive immunotherapy attenuated both Tau pathology and cognitive deficits. These findings align with prior reports highlighting the role of Tau truncation in tauopathy pathogenesis, particularly in AD [42].

While many studies have focused on C-terminally truncated Tau species, N-terminal truncations remain comparatively underexplored [8, 43]. Our findings significantly expand current knowledge by identifying an N-terminal truncated Tau variant that is not only novel in sequence but also exhibits an N-α-acetylation, a post-translational modification so far not reported for Tau. To date, the generation of truncated Tau species is traditionally attributed to proteolytic cleavage of FL-Tau [43], resulting in fragments which could then contribute to Tau aggregation and neurotoxicity [7, 8, 42, 44–48]. However, a collection of evidence supports the hypothesis that AcMet11-Tau arises via a non-canonical translation initiation mechanism. First, the AUG codon for Met11 is embedded within an optimal Kozak consensus sequence [49], supporting its role as a bona fide alternative translation starting site. Second, Met11 residue is likely acetylated in a co-translational manner by N-acetyl transferase B, an enzyme that is active only when recruited by translating ribosomes [50]. Such data suggest de novo synthesis rather than post-translational cleavage. Third, our analyses of published ribosome profiling data [51] indicate the presence of initiating ribosomes on the Met11 codon, reinforcing its role as an alternative translation initiation site. In line, we have recently shown with rabbit reticulocyte lysates and HEK293T extracts that translation of Met11-Tau occurs from in vitro-synthesized tau mRNA transcripts [52]. This mechanism is particularly intriguing, as dysregulated translation initiation is increasingly recognized in human diseases [53, 54]. For instance, the recognition of alternative translation initiation sites (ATIS) by the ribosome producing N-terminally truncated variants has been linked to pathological outcomes [55–57], such as insulin resistance resulting from the recognition of an ATIS at the Met-14 in Caveolin-2β [58]. Our discovery of AcMet11-Tau suggests that non-canonical translational regulation may contribute to AD pathogenesis, warranting further investigation into the molecular mechanisms governing this process.

Moreover, alteration of the N-terminal acetylation pattern is also reported to be involved in human diseases [21, 22, 59]. One key critical role of this prevalent protein modification is the regulation of protein stability; acetylation can protect proteins from degradation, thereby influencing protein half-life [60]. Additionally, this modification has been shown to affect protein conformation and folding [21]. For instance, in proteins associated with neurodegenerative diseases, such as α-synuclein, N-terminal acetylation influences their propensity to misfold and aggregate [59]. For AcMet11-Tau, the functional consequences of N-α-acetylation remain to be fully elucidated. It is plausible that this modification enhances protein stability, protecting AcMet11-Tau from degradation and prolonging its half-life, or alters its folding, thereby promoting aggregation and seeding of FL-Tau as previously described [61].

The early detection of AcMet11-Tau in both transgenic mice and AD patient brains suggests an initiating role of AcMet11-Tau in disease progression. Our in vivo functional experiments further demonstrate that expression of AcMet11-Tau potentiates Tau pathology, while its targeted neutralization mitigates Tau pathology and cognitive decline. These results are consistent with prior reports, based on either expression or passive immunotherapy, showing that N-terminally truncated Tau variants contribute to Tau pathology development in vivo [8, 62, 63]. We note, however, that the lentiviral expression experiments were performed with relatively small group sizes, and that the present study was designed as an exploratory investigation to assess the functional relevance of AcMet11-Tau rather than to provide definitive quantitative correlation between AcMet11-Tau expression levels and AT100-positive Tau pathology. Future work, including larger cohorts and quantitative assays, will be essential to clarify this relationship and to determine whether AcMet11-Tau levels correlate with pathological burden or disease progression. Importantly, the significance of AcMet11-Tau in this study is supported by its biochemical characterization, its selective association with AD pathology, and its functional impact on Tau aggregation and cognitive deficits in vivo. As previously demonstrated for other seeding-competent Tau species [64, 65], pathological relevance is not solely determined by abundance. Future studies will focus on directly assessing the seeding activity of AcMet11-Tau using sensitive functional assays [66, 67].

From a translational perspective, AcMet11-Tau holds dual potential as a biomarker and a therapeutic target. Tau immunotherapy is a promising avenue for AD treatment [68–70]. Even the mechanisms underlying the beneficial effect of Tau-based immunotherapy are still to be determined, passive immunotherapies in rodent models of tauopathies have shown that monoclonal antibodies injected via the systemic pathway can cross the blood–brain barrier (BBB) and bind to Tau-targeted proteins [71, 72]. In the present study, two delivery paradigms that may engage different mechanisms of action were employed. Direct intracerebral administration was used as an exploratory approach to determine whether local neutralization of AcMet11-Tau could directly influence Tau pathology in vivo, independently of BBB constraints. In contrast, systemic administration was designed to assess the translational feasibility of targeting AcMet11-Tau under clinically relevant conditions, despite limited brain exposure. In line, following systemic administration of the 2H2D11 antibody, we observed robust peripheral exposure, as evidenced by its stable detection in plasma. However, we were unable to reliably detect the antibody in brain tissue, suggesting that only a very small fraction of circulating 2H2D11 reaches the brain, likely below the detection threshold of our assays. This observation is consistent with previous reports indicating limited BBB penetration of systemically administered IgG antibodies [73, 74]. Alternative delivery strategies should be explored. However, a major challenge lies in selectively targeting pathological Tau species without disrupting normal Tau function. Our findings address this challenge: AcMet11-Tau is exclusively detected in pathological contexts, and our specific antibody, 2H2D11, effectively binds this new variant without cross-reacting with physiological Tau. Importantly, the present work is intended as a proof of concept demonstrating the therapeutic relevance of selectively targeting a disease-specific Tau species, and does not aim to compare the efficacy of 2H2D11 with other Tau-directed antibodies currently under development. Accordingly, the high specificity of 2H2D11 together with the observed reduction in Tau pathology and associated cognitive deficits, supports the therapeutic relevance of AcMet11-Tau targeting, even in the context of limited antibody brain penetration.

## Conclusion

This study identifies a previously overlooked AD-relevant Tau species. Through its characterization, selective enrichment in AD brain tissue, and functional impact on Tau pathology, our findings support AcMet11-Tau as a biologically relevant pathological Tau variant with potential translational value. The development and validation of a specific monoclonal antibody enabled the detection and targeting of this species, providing initial in *vivo* evidence supporting the feasibility of AcMet11-Tau-directed immunotherapy to modulate Tau pathology and partially associated neuroinflammatory markers.

Future studies will be required to evaluate the potential of AcMet11-Tau as a fluid or imaging biomarker, notably through the development of ultrasensitive assays in line with revised AD diagnostic criteria [75]. It will also be important to demonstrate the selective association of AcMet11-Tau with AD by analysing more primary tauopathies. Notably, studies in larger and well-characterized human post-mortem cohorts will be required to validate the immunohistochemical characterization of AcMet11-Tau pathology. In parallel, additional research is needed to elucidate the molecular mechanisms underlying the production and pathogenicity of AcMet11-Tau, as it could also represent a therapeutic target. Finally, the safety and efficacy of AcMet11-Tau-targeted immunotherapies will need to be evaluated in translational and clinical settings. In this context, studies using BBB-penetrant antibody formats or alternative delivery strategies [76] will be necessary to directly assess central target engagement and translational applicability.

## Supplementary Information

Additional file 1. **Fig. S1**. Experimental design of passive immunotherapy. **Fig. S2**. Immunohistochemical detection of AcMet11-Tau in hippocampal human brain sections. **Fig. S3**. Biochemical characterization of Tau pathology in PSP brain samples. **Fig. S4**. Characterization of Tau pathology regarding Braak stages and relationship between AcMet11-Tau and pSer396 ELISA measurements. **Fig. S5**. Validation of lentiviral vector batches in primary neuronal cells. **Fig. S6**. Antibody titer and mouse body weight during immunization. **Fig. S7**. Locomotor activity and anxiety-like behavior. **Table S1**. Summary of human brain tissues. **Table S2**. Antibodies used in this study. **Table S3**. Primers used in qPCR. Additional file 2. Uncropped Western blots.

## Data Availability

All data and methods used in this study are available in the main text or the supplementary materials. The data generated during the current study are available as preprint in the bioRxiv: https://www.biorxiv.org/cgi/content/short/2025.10.03.680200v1. The mass spectrometry proteomics data have been deposited to the ProteomeXchange Consortium (http://proteomecentral.proteomexchange.org) via the PRIDE [33] partner repository with the dataset identifier PXD072580 and 10.6019/PXD072580. The VH and VL sequences of 2H2D11 antibody are available on request. Cell and animal models, as well as the antibodies generated in this study are available by contacting LB under MTA with Inserm.

## Abbreviations

AcMet11: N-terminally acetylated methionine 11
AD: Alzheimer’s disease
ATIS: Alternative translation initiation site
BA: Brodmann area
EPM: Elevated Plus Maze
FL-Tau: Full-length Tau
IHC: Immunohistochemistry
IP: Intraperitonial injection
KLH: Keyhole limpet hemocyanin
LC-MS/MS: Liquid chromatography-tandem mass spectrometry
Lv: Lentiviral vector
NAT: Nα-terminal acetyltransferase
NFD: Neurofibrillary degeneration
NFT: Neurofibrillary tangles
OD: Optical density
PiD: Pick’s disease
PSP: Progressive supranuclear palsy
WT: Wild-type

## References

[1] Caillet-Boudin M-L, Buée L, Sergeant N, Lefebvre B. Regulation of human MAPT gene expression. Mol Neurodegener. 2015;10:28. 26170022 10.1186/s13024-015-0025-8 PMC4499907

[2] Limorenko G, Lashuel HA. Revisiting the grammar of Tau aggregation and pathology formation: how new insights from brain pathology are shaping how we study and target Tauopathies. Chem Soc Rev. 2022;51:513–65. 34889934 10.1039/d1cs00127b

[3] Braak H, Braak E. Staging of Alzheimer’s disease-related neurofibrillary changes. Neurobiol Aging. 1995;16:271–8 (discussion 278-284). 7566337 10.1016/0197-4580(95)00021-6

[4] Duyckaerts C, Colle MA, Dessi F, Piette F, Hauw JJ. Progression of Alzheimer histopathological changes. Acta Neurol Belg. 1998;98:180–5. 9686277

[5] Delacourte A, David JP, Sergeant N, Buée L, Wattez A, Vermersch P, et al. The biochemical pathway of neurofibrillary degeneration in aging and Alzheimer’s disease. Neurology. 1999;52:1158–65. 10214737 10.1212/wnl.52.6.1158

[6] Zilka N, Kovacech B, Barath P, Kontsekova E, Novák M. The self-perpetuating tau truncation circle. Biochem Soc Trans. 2012;40:681–6. 22817716 10.1042/BST20120015

[7] Quinn JP, Corbett NJ, Kellett KAB, Hooper NM. Tau proteolysis in the pathogenesis of tauopathies: neurotoxic fragments and novel biomarkers. J Alzheimers Dis. 2018;63:13–33. 29630551 10.3233/JAD-170959 PMC5900574

[8] Amadoro G, Latina V, Corsetti V, Calissano P. N-terminal tau truncation in the pathogenesis of Alzheimer’s disease (AD): developing a novel diagnostic and therapeutic approach. Biochim Biophys Acta Mol Basis Dis. 2020;1866(3):165584. 31676377 10.1016/j.bbadis.2019.165584

[9] Zhou Y, Shi J, Chu D, Hu W, Guan Z, Gong C-X, et al. Relevance of phosphorylation and truncation of tau to the etiopathogenesis of alzheimer’s disease. Front Aging Neurosci. 2018;10:27. 29472853 10.3389/fnagi.2018.00027 PMC5810298

[10] da Costa PJ, Hamdane M, Buée L, Martin F. Tau mRNA metabolism in neurodegenerative diseases: a tangle journey. Biomedicines. 2022;10:241. 35203451 10.3390/biomedicines10020241 PMC8869323

[11] Derisbourg M, Leghay C, Chiappetta G, Fernandez-Gomez F-J, Laurent C, Demeyer D, et al. Role of the Tau N-terminal region in microtubule stabilization revealed by new endogenous truncated forms. Sci Rep. 2015;5:9659. 25974414 10.1038/srep09659 PMC4431475

[12] Hayashi S, Toyoshima Y, Hasegawa M, Umeda Y, Wakabayashi K, Tokiguchi S, et al. Late-onset frontotemporal dementia with a novel exon 1 (Arg5His) Tau gene mutation. Ann Neurol. 2002;51:525–30. 11921059 10.1002/ana.10163

[13] Poorkaj P, Muma NA, Zhukareva V, Cochran EJ, Shannon KM, Hurtig H, et al. An R5L Tau mutation in a subject with a progressive supranuclear palsy phenotype. Ann Neurol. 2002;52:511–6. 12325083 10.1002/ana.10340

[14] Magnani E, Fan J, Gasparini L, Golding M, Williams M, Schiavo G, et al. Interaction of Tau protein with the dynactin complex. EMBO J. 2007;26:4546–54. 17932487 10.1038/sj.emboj.7601878 PMC2063488

[15] Carmel G, Mager EM, Binder LI, Kuret J. The structural basis of monoclonal antibody Alz50’s selectivity for Alzheimer’s disease pathology. J Biol Chem. 1996;271:32789–95. 8955115 10.1074/jbc.271.51.32789

[16] Jeganathan S, von Bergen M, Brutlach H, Steinhoff H-J, Mandelkow E. Global hairpin folding of Tau in solution. Biochemistry. 2006;45:2283–93. 16475817 10.1021/bi0521543

[17] Jeganathan S, Hascher A, Chinnathambi S, Biernat J, Mandelkow E-M, Mandelkow E. Proline-directed pseudo-phosphorylation at AT8 and PHF1 epitopes induces a compaction of the paperclip folding of Tau and generates a pathological (MC-1) conformation. J Biol Chem. 2008;283:32066–76. 18725412 10.1074/jbc.M805300200

[18] Polevoda B, Sherman F. Composition and function of the eukaryotic N-terminal acetyltransferase subunits. Biochem Biophys Res Commun. 2003;308:1–11. 12890471 10.1016/s0006-291x(03)01316-0

[19] Arnesen T, Van Damme P, Polevoda B, Helsens K, Evjenth R, Colaert N, et al. Proteomics analyses reveal the evolutionary conservation and divergence of N-terminal acetyltransferases from yeast and humans. Proc Natl Acad Sci U S A. 2009;106:8157–62. 19420222 10.1073/pnas.0901931106 PMC2688859

[20] Drazic A, Myklebust LM, Ree R, Arnesen T. The world of protein acetylation. Biochim Biophys Acta. 2016;1864:1372–401. 27296530 10.1016/j.bbapap.2016.06.007

[21] Calis S, Gevaert K. The role of Nα‐terminal acetylation in protein conformation. FEBS J. 2025;292:453–67. 38923676 10.1111/febs.17209

[22] McTiernan N, Kjosås I, Arnesen T. Illuminating the impact of N-terminal acetylation: from protein to physiology. Nat Commun. 2025;16:703. 39814713 10.1038/s41467-025-55960-5 PMC11735805

[23] Braak H, Thal DR, Ghebremedhin E, Del Tredici K. Stages of the pathologic process in Alzheimer disease: age categories from 1 to 100 years. J Neuropathol Exp Neurol. 2011;70:960–9. 22002422 10.1097/NEN.0b013e318232a379

[24] Schindowski K, Bretteville A, Leroy K, Bégard S, Brion J-P, Hamdane M, et al. Alzheimer’s disease-like tau neuropathology leads to memory deficits and loss of functional synapses in a novel mutated tau transgenic mouse without any motor deficits. Am J Pathol. 2006;169:599–616. 16877359 10.2353/ajpath.2006.060002 PMC1698785

[25] Van der Jeugd A, Vermaercke B, Derisbourg M, Lo AC, Hamdane M, Blum D, et al. Progressive age-related cognitive decline in tau mice. J Alzheimers Dis. 2013;37:777–88. 23948912 10.3233/JAD-130110

[26] Laurent C, Dorothée G, Hunot S, Martin E, Monnet Y, Duchamp M, et al. Hippocampal T cell infiltration promotes neuroinflammation and cognitive decline in a mouse model of tauopathy. Brain. 2017;140:184–200. 27818384 10.1093/brain/aww270 PMC5382942

[27] Leroy K, Bretteville A, Schindowski K, Gilissen E, Authelet M, De Decker R, et al. Early axonopathy preceding neurofibrillary tangles in mutant tau transgenic mice. Am J Pathol. 2007;171:976–92. 17690183 10.2353/ajpath.2007.070345 PMC1959508

[28] Mailliot C, Bussière T, Hamdane M, Sergeant N, Caillet M-L, Delacourte A, et al. Pathological tau phenotypes: the weight of mutations, polymorphisms, and differential neuronal vulnerabilities. Ann N Y Acad Sci. 2000;920:107–14. 11193138 10.1111/j.1749-6632.2000.tb06911.x

[29] Deglon N, Tseng JL, Bensadoun J-C, Zurn AD, Arsenijevic Y, Almeida LPD, et al. Self-inactivating lentiviral vectors with enhanced transgene expression as potential gene transfer system in Parkinson’s disease. Human Gene Therapy. 2000;11:179–90. 10646649 10.1089/10430340050016256

[30] Caillierez R, Bégard S, Lécolle K, Deramecourt V, Zommer N, Dujardin S, et al. Lentiviral delivery of the human wild-type tau protein mediates a slow and progressive neurodegenerative tau pathology in the rat brain. Mol Ther. 2013;21:1358. 23609018 10.1038/mt.2013.66 PMC3702115

[31] Hamdane M, Sambo A-V, Delobel P, Bégard S, Violleau A, Delacourte A, et al. Mitotic-like tau phosphorylation by p25-Cdk5 kinase complex. J Biol Chem. 2003;278:34026–34. 12826674 10.1074/jbc.M302872200

[32] Denechaud M, Geurs S, Comptdaer T, Bégard S, Garcia-Núñez A, Pechereau L-A, et al. Tau promotes oxidative stress-associated cycling neurons in S phase as a pro-survival mechanism: possible implication for Alzheimer’s disease. Prog Neurobiol. 2023;223:102386. 36481386 10.1016/j.pneurobio.2022.102386

[33] Perez-Riverol Y, Bandla C, Kundu DJ, Kamatchinathan S, Bai J, Hewapathirana S, et al. The PRIDE database at 20 years: 2025 update. Nucleic Acids Res. 2025;53:D543–53. 39494541 10.1093/nar/gkae1011 PMC11701690

[34] Pandey S. Hybridoma technology for production of monoclonal antibodies. Int J Pharm Sci Rev Res. 2010;1:88–94.

[35] Trebak M, Chong JM, Herlyn D, Speicher DW. Efficient laboratory-scale production of monoclonal antibodies using membrane-based high-density cell culture technology. J Immunol Methods. 1999;230:59–70. 10594354 10.1016/s0022-1759(99)00122-2

[36] Brito LA, Singh M. Acceptable levels of endotoxin in vaccine formulations during preclinical research. J Pharm Sci. 2011;100(1):34–7. 20575063 10.1002/jps.22267

[37] Agadjanyan MG, Zagorski K, Petrushina I, Davtyan H, Kazarian K, Antonenko M, et al. Humanized monoclonal antibody armanezumab specific to N-terminus of pathological tau: characterization and therapeutic potency. Mol Neurodegener. 2017;12:33. 28472993 10.1186/s13024-017-0172-1 PMC5418694

[38] Weaver CL, Espinoza M, Kress Y, Davies P. Conformational change as one of the earliest alterations of tau in Alzheimer’s disease. Neurobiol Aging. 2000;21:719–27. 11016541 10.1016/s0197-4580(00)00157-3

[39] Augustinack JC, Schneider A, Mandelkow E-M, Hyman BT. Specific tau phosphorylation sites correlate with severity of neuronal cytopathology in Alzheimer’s disease. Acta Neuropathol. 2002;103:26–35. 11837744 10.1007/s004010100423

[40] Laurent C, Burnouf S, Ferry B, Batalha VL, Coelho JE, Baqi Y, et al. A2A adenosine receptor deletion is protective in a mouse model of Tauopathy. Mol Psychiatry. 2016;21:97–107. 25450226 10.1038/mp.2014.151

[41] Carvalho K, Faivre E, Pietrowski MJ, Marques X, Gomez-Murcia V, Deleau A, et al. Exacerbation of C1q dysregulation, synaptic loss and memory deficits in tau pathology linked to neuronal adenosine A2A receptor. Brain. 2019;142:3636–54. 31599329 10.1093/brain/awz288 PMC6821333

[42] Novak P, Cehlar O, Skrabana R, Novak M. Tau conformation as a target for disease-modifying therapy: the role of truncation. J Alzheimers Dis. 2018;64:S535–46. 29865059 10.3233/JAD-179942

[43] Chu D, Yang X, Wang J, Zhou Y, Gu J-H, Miao J, et al. Tau truncation in the pathogenesis of Alzheimer’s disease: a narrative review. Neural Regen Res. 2024;19:1221–32. 37905868 10.4103/1673-5374.385853 PMC11467920

[44] Rissman RA, Poon WW, Blurton-Jones M, Oddo S, Torp R, Vitek MP, et al. Caspase-cleavage of tau is an early event in Alzheimer disease tangle pathology. J Clin Invest. 2004;114:121–30. 15232619 10.1172/JCI20640 PMC437967

[45] Basurto-Islas G, Luna-Muñoz J, Guillozet-Bongaarts AL, Binder LI, Mena R, García-Sierra F. Accumulation of aspartic acid421- and glutamic acid391-cleaved tau in neurofibrillary tangles correlates with progression in Alzheimer disease. J Neuropathol Exp Neurol. 2008;67:470–83. 18431250 10.1097/NEN.0b013e31817275c7 PMC4699801

[46] de Calignon A, Fox LM, Pitstick R, Carlson GA, Bacskai BJ, Spires-Jones TL, et al. Caspase activation precedes and leads to tangles. Nature. 2010;464:1201–4. 20357768 10.1038/nature08890 PMC3091360

[47] Zhang Z, Song M, Liu X, Kang SS, Kwon I-S, Duong DM, et al. Cleavage of tau by asparagine endopeptidase mediates the neurofibrillary pathology in Alzheimer’s disease. Nat Med. 2014;20:1254–62. 25326800 10.1038/nm.3700 PMC4224595

[48] Zhao X, Kotilinek LA, Smith B, Hlynialuk C, Zahs K, Ramsden M, et al. Caspase-2 cleavage of tau reversibly impairs memory. Nat Med. 2016;22:1268–76. 27723722 10.1038/nm.4199

[49] Kozak M. At least six nucleotides preceding the AUG initiator codon enhance translation in mammalian cells. J Mol Biol. 1987;196:947–50. 3681984 10.1016/0022-2836(87)90418-9

[50] Starheim KK, Gevaert K, Arnesen T. Protein N-terminal acetyltransferases: when the start matters. Trends Biochem Sci. 2012;37:152–61. 22405572 10.1016/j.tibs.2012.02.003

[51] Michel AM, Fox G, Kiran AM, De Bo C, Oconnor PBF, Heaphy SM, et al. GWIPS-viz: development of a ribo-seq genome browser. Nucleic Acids Res. 2014;42:859–64. 10.1093/nar/gkt1035 PMC3965066 24185699

[52] da Costa PJ, Perret A, Buée L, Hamdane M, Martin F. FTLD-MAPT mutations and short 5′UTR Tau mRNAs increase Tau translation. NAR Mol Med. 2024;1:ugae023. 41257254 10.1093/narmme/ugae023 PMC12430014

[53] Tahmasebi S, Khoutorsky A, Mathews MB, Sonenberg N. Translation deregulation in human disease. Nat Rev Mol Cell Biol. 2018;19:791–807. 30038383 10.1038/s41580-018-0034-x

[54] James CC, Smyth JW. Alternative mechanisms of translation initiation: an emerging dynamic regulator of the proteome in health and disease. Life Sci. 2018;212:138–44. 30290184 10.1016/j.lfs.2018.09.054 PMC6345546

[55] Yin Y, Stephen CW, Luciani MG, Fåhraeus R. p53 stability and activity is regulated by Mdm2-mediated induction of alternative p53 translation products. Nat Cell Biol. 2002;4:462–7. 12032546 10.1038/ncb801

[56] Jaud M, Philippe C, Van Den Berghe L, Ségura C, Mazzolini L, Pyronnet S, et al. The PERK branch of the unfolded protein response promotes DLL4 expression by activating an alternative translation mechanism. Cancers (Basel). 2019;11:142. 30691003 10.3390/cancers11020142 PMC6406545

[57] Bogaert A, Fernandez E, Gevaert K. N-terminal proteoforms in human disease. Trends Biochem Sci. 2020;45:308–20. 32001092 10.1016/j.tibs.2019.12.009

[58] Kwon H, Jang D, Choi M, Lee J, Jeong K, Pak Y. Alternative translation initiation of Caveolin-2 desensitizes insulin signaling through dephosphorylation of insulin receptor by PTP1B and causes insulin resistance. Biochim Biophys Acta (BBA). 2018;1864:2169–82. 10.1016/j.bbadis.2018.03.022 29604334

[59] Iyer A, Sidhu A, Subramaniam V. How important is the N-terminal acetylation of alpha-synuclein for its function and aggregation into amyloids? Front Neurosci. 2022;16:1003997. 36466161 10.3389/fnins.2022.1003997 PMC9709446

[60] Varland S, Silva RD, Kjosås I, Faustino A, Bogaert A, Billmann M, et al. N-terminal acetylation shields proteins from degradation and promotes age-dependent motility and longevity. Nat Commun. 2023;14:6774. 37891180 10.1038/s41467-023-42342-y PMC10611716

[61] Ozcelik S, Sprenger F, Skachokova Z, Fraser G, Abramowski D, Clavaguera F, et al. Co-expression of truncated and full-length tau induces severe neurotoxicity. Mol Psychiatry. 2016;21:1790–8. 26830137 10.1038/mp.2015.228 PMC5116481

[62] Borreca A, Latina V, Corsetti V, Middei S, Piccinin S, Della Valle F, et al. AD-related N-terminal truncated tau is sufficient to recapitulate in vivo the early perturbations of human neuropathology: implications for immunotherapy. Mol Neurobiol. 2018;55:8124–53. 29508283 10.1007/s12035-018-0974-3

[63] Corsetti V, Borreca A, Latina V, Giacovazzo G, Pignataro A, Krashia P, et al. Passive immunotherapy for N-truncated tau ameliorates the cognitive deficits in two mouse Alzheimer’s disease models. Brain Commun. 2020;2:fcaa039. 32954296 10.1093/braincomms/fcaa039 PMC7425324

[64] DeVos SL, Corjuc BT, Oakley DH, Nobuhara CK, Bannon RN, Chase A, et al. Synaptic tau seeding precedes tau pathology in human Alzheimer’s disease brain. Front Neurosci. 2018;12:267. 29740275 10.3389/fnins.2018.00267 PMC5928393

[65] Mirbaha H, Chen D, Mullapudi V, Terpack SJ, White CL, Joachimiak LA, et al. Seed-competent tau monomer initiates pathology in a tauopathy mouse model. J Biol Chem. 2022;298:102163. 35750209 10.1016/j.jbc.2022.102163 PMC9307951

[66] Holmes BB, Furman JL, Mahan TE, Yamasaki TR, Mirbaha H, Eades WC, et al. Proteopathic tau seeding predicts tauopathy in vivo. Proc Natl Acad Sci U S A. 2014;111:E4376-4385. 25261551 10.1073/pnas.1411649111 PMC4205609

[67] Frey B, Holzinger D, Taylor K, Ehrnhoefer DE, Striebinger A, Biesinger S, et al. Tau seed amplification assay reveals relationship between seeding and pathological forms of tau in Alzheimer’s disease brain. Acta Neuropathol Commun. 2023;11:181. 37964332 10.1186/s40478-023-01676-w PMC10644662

[68] Bittar A, Bhatt N, Kayed R. Advances and considerations in AD tau-targeted immunotherapy. Neurobiol Dis. 2020;134:104707. 31841678 10.1016/j.nbd.2019.104707 PMC6980703

[69] Sandusky-Beltran LA, Sigurdsson EM. Tau immunotherapies: lessons learned, current status and future considerations. Neuropharmacology. 2020;175:108104. 32360477 10.1016/j.neuropharm.2020.108104 PMC7492435

[70] Congdon EE, Ji C, Tetlow AM, Jiang Y, Sigurdsson EM. Tau-targeting therapies for Alzheimer disease: current status and future directions. Nat Rev Neurol. 2023;19:715–36. 37875627 10.1038/s41582-023-00883-2 PMC10965012

[71] Ittner A, Bertz J, Suh LS, Stevens CH, Götz J, Ittner LM. Tau‐targeting passive immunization modulates aspects of pathology in tau transgenic mice. J Neurochem. 2015;132(1):135–45. 25041093 10.1111/jnc.12821

[72] Sigurdsson EM. Tau immunotherapy. Neurodegener Dis. 2016;16:34–8. 26551002 10.1159/000440842 PMC4777344

[73] Nerenberg ST, Prasad R. Radioimmunoassays for Ig classes G, A, M, D, and E in spinal fluids: normal values of different age groups. J Lab Clin Med. 1975;86:887–98. 1185045

[74] Pardridge WM. The blood-brain barrier: bottleneck in brain drug development. NeuroRx. 2005;2:3–14. 15717053 10.1602/neurorx.2.1.3 PMC539316

[75] Jack CR, Andrews JS, Beach TG, Buracchio T, Dunn B, Graf A, et al. Revised criteria for diagnosis and staging of Alzheimer’s disease: Alzheimer’s Association Workgroup. Alzheimers Dement. 2024;20:5143–69. 38934362 10.1002/alz.13859 PMC11350039

[76] Manoutcharian K, Perez-Garmendia R, Gevorkian G. Recombinant antibody fragments for neurodegenerative diseases. Curr Neuropharmacol. 2017;15:779–88. 27697033 10.2174/1570159X01666160930121647 PMC5771054

