# Supplementary material for: N-terminally acetylated Met11-Tau: a new pathological truncated Tau species with functional relevance in Alzheimer's disease
Source: Transl Neurodegener. 2026 Apr 27;15:19. doi: 10.1186/s40035-026-00550-8 (PMC13123110; doi:10.1186/s40035-026-00550-8)
Supplement: Supplementary file 1 — Additional file 1. Fig. S1. Experimental design of passive immunotherapy. Fig. S2. Immunohistochemical detection of AcMet11-Tau in hippocampal human brain sections. Fig. S3. Biochemical characterization of Tau pathology in PSP brain samples. Fig. S4. Characterization of Tau pathology regarding Braak stages and relationship between AcMet11-Tau and pSer396 ELISA measurements. Fig. S5. Validation of lentiviral vector batches in primary neuronal cells. Fig. S6. Antibody titer and mouse body weight during immunization. Fig. S7. Locomotor activity and anxiety-like behavior. Table S1. Summary of human brain tissues. Table S2. Antibodies used in this study. Table S3. Primers used in qPCR. Additional file 2. Uncropped Western blots. [file 40035_2026_550_MOESM1_ESM.pdf]

Fig. 1d. AcMet11-Tau

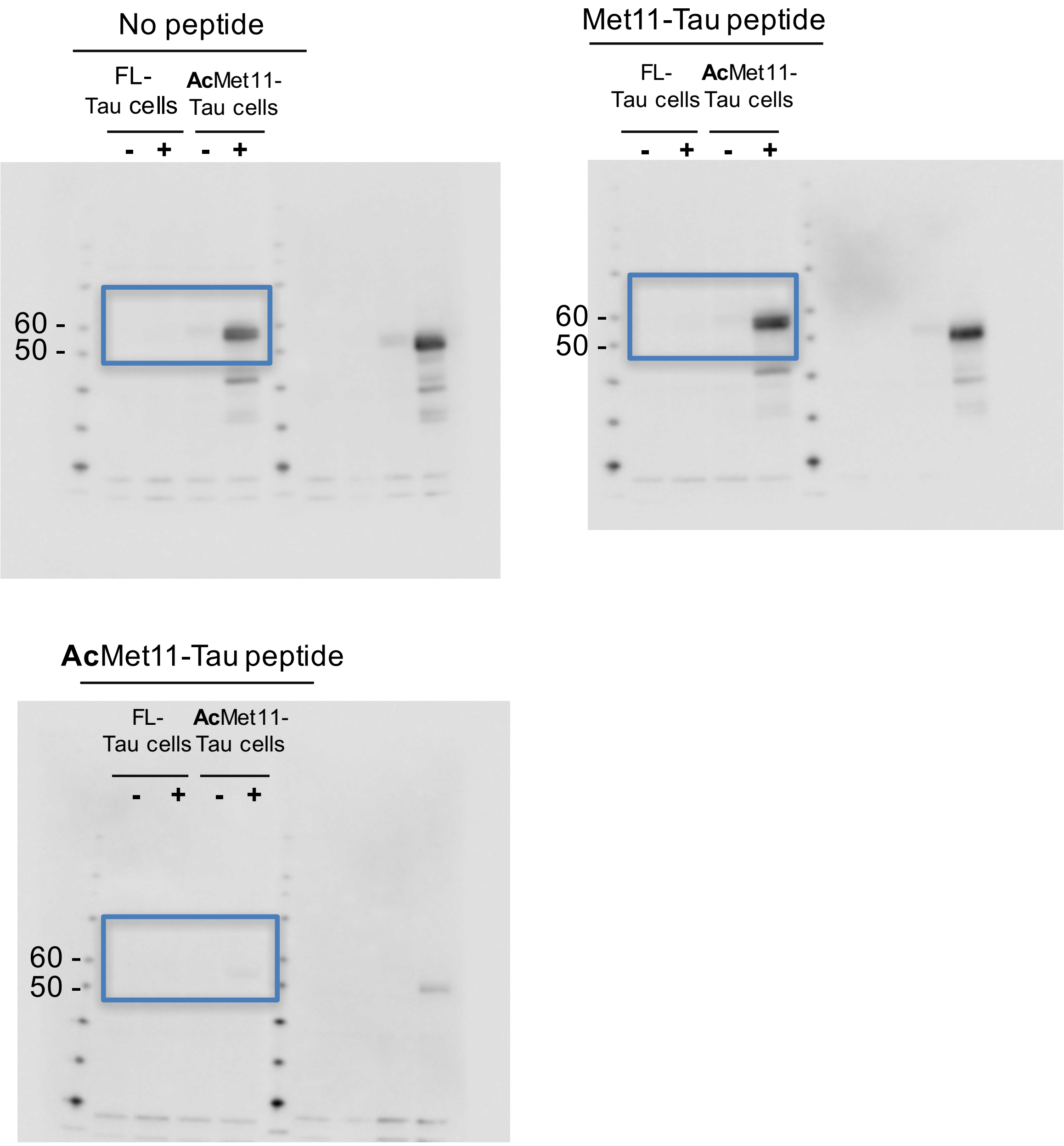

Fig. 1d. Total Tau

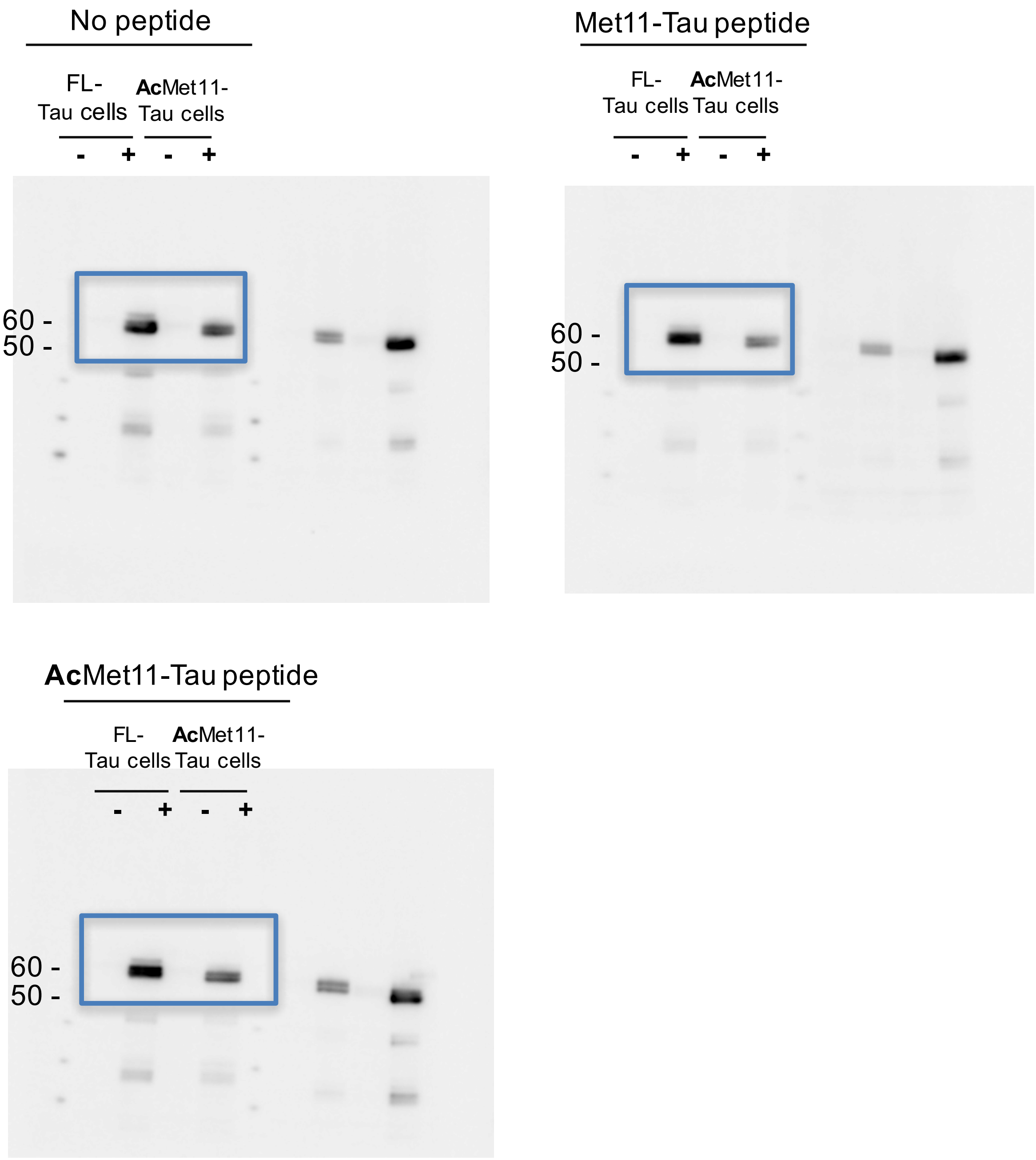

Fig. 1d. NSE

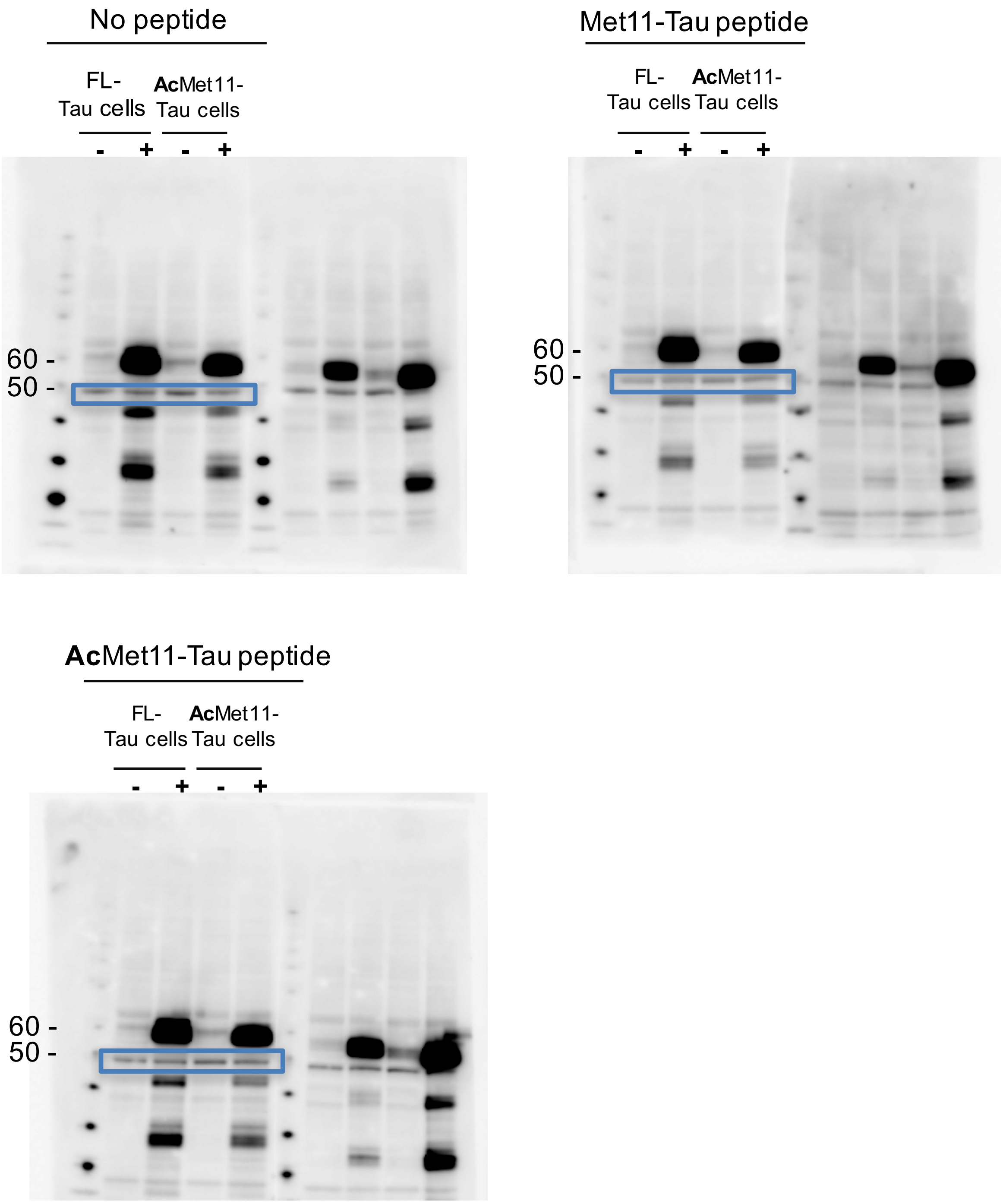
No peptide
FL-
Tau cells
AcMet11-
Tau cells
- + - +
60 -
50 -
Met11-Tau peptide
FL-
Tau cells
AcMet11-
Tau cells
- + - +
60 -
50 -
AcMet11-Tau peptide
FL-
Tau cells
AcMet11-
Tau cells
- + - +
60 -
50 -

Fig. S2. Tau-pSer396

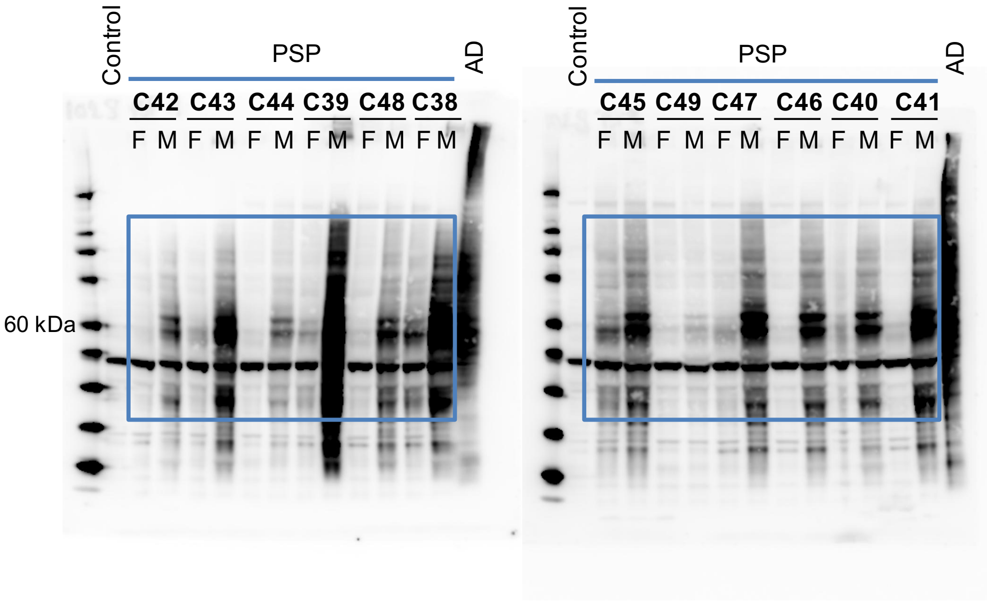

Fig. S2. β-actin

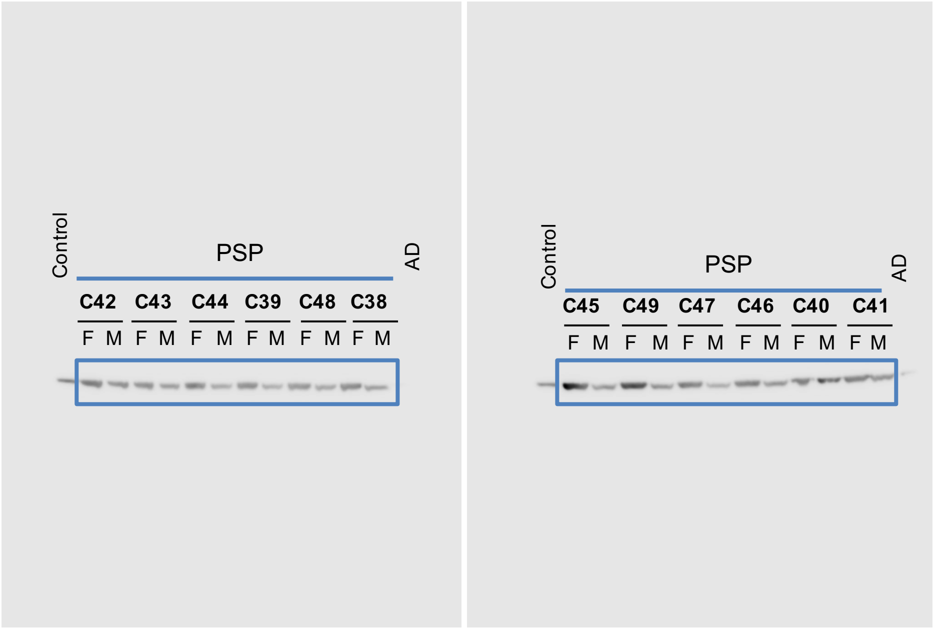

Fig. S3. Tau-pSer396 and GAPDH (BA-38)

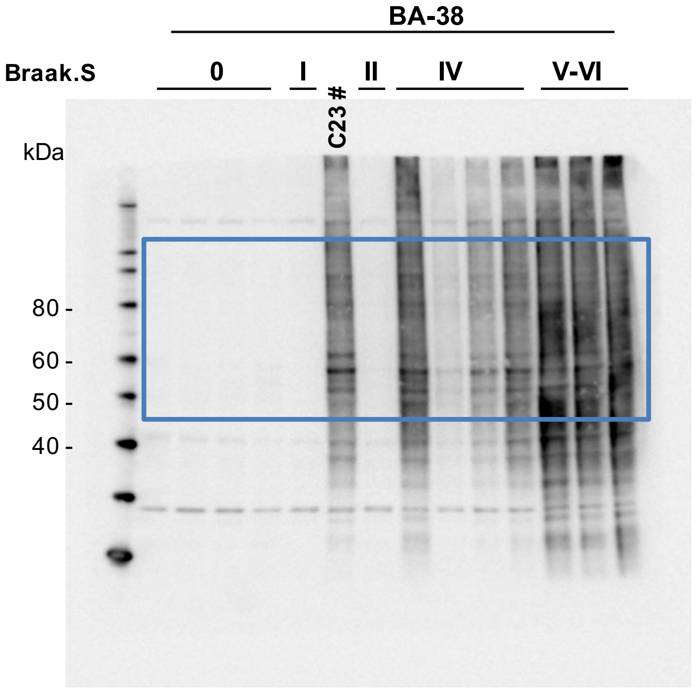

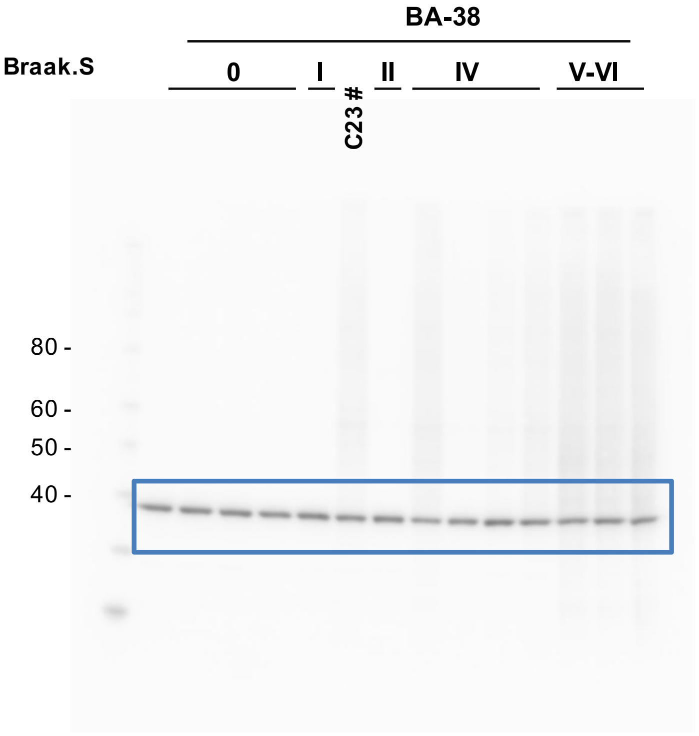

Fig. S3. Total Tau and GAPDH (BA-38)

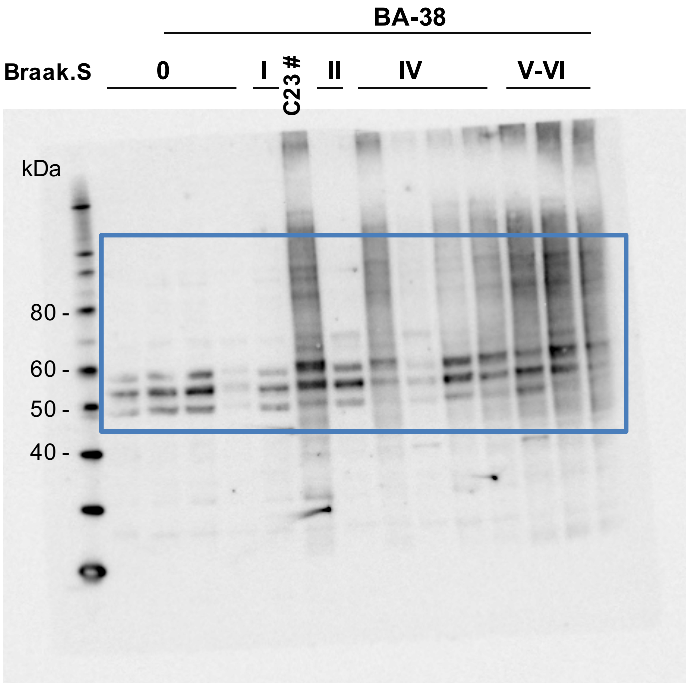

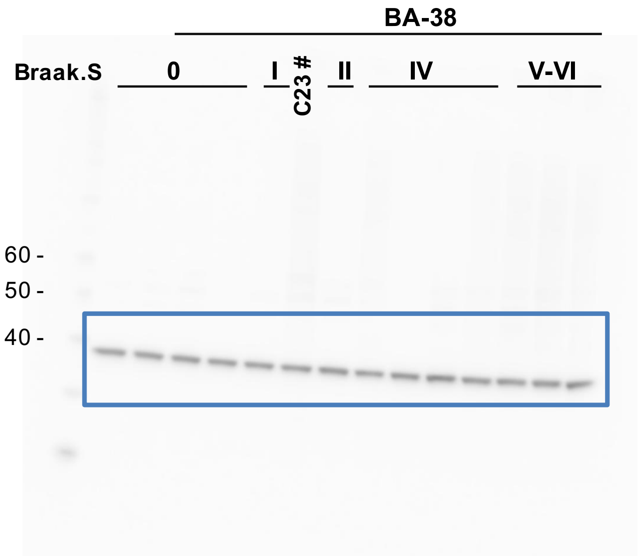

Fig. S3. Tau-pSer396 and GAPDH (BA-10)

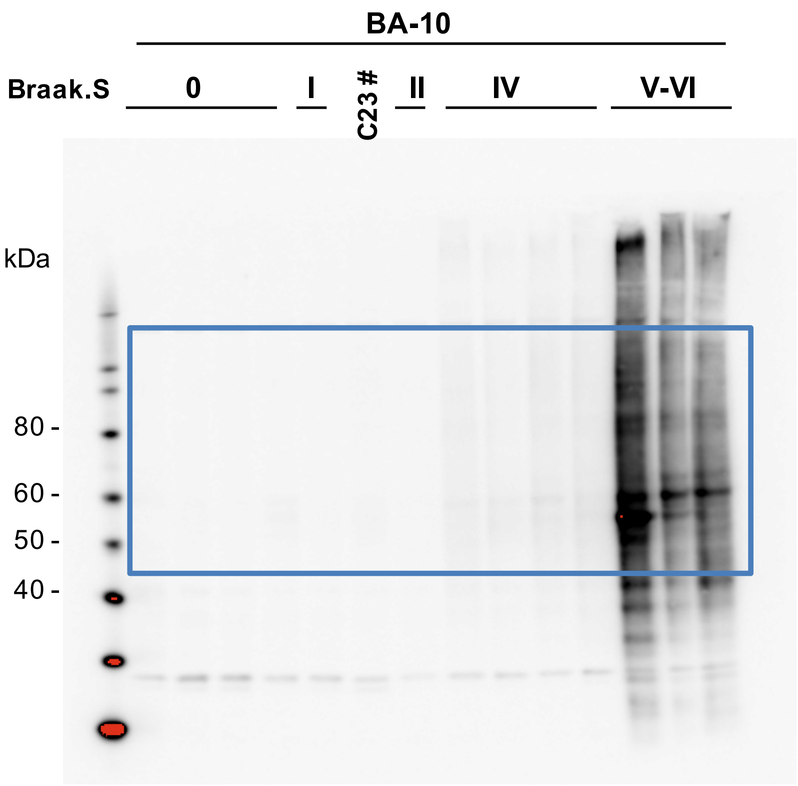

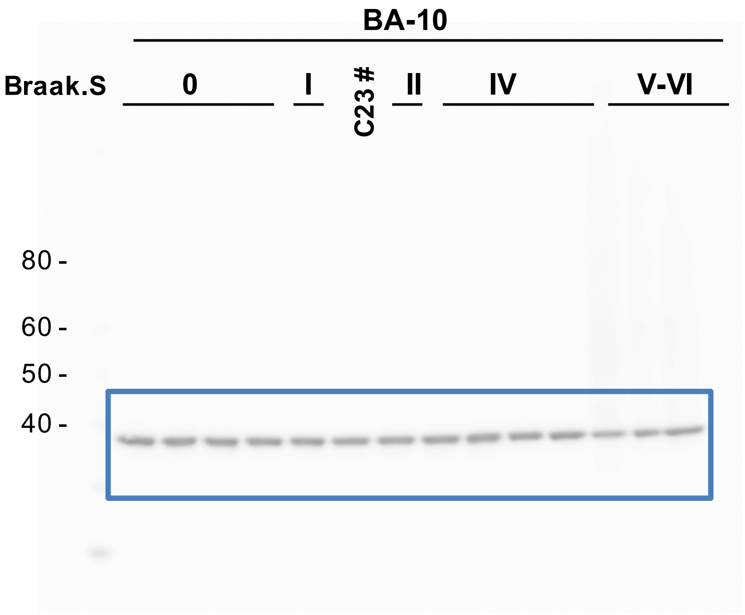

Fig. S3. Total Tau and GAPDH (BA-10)

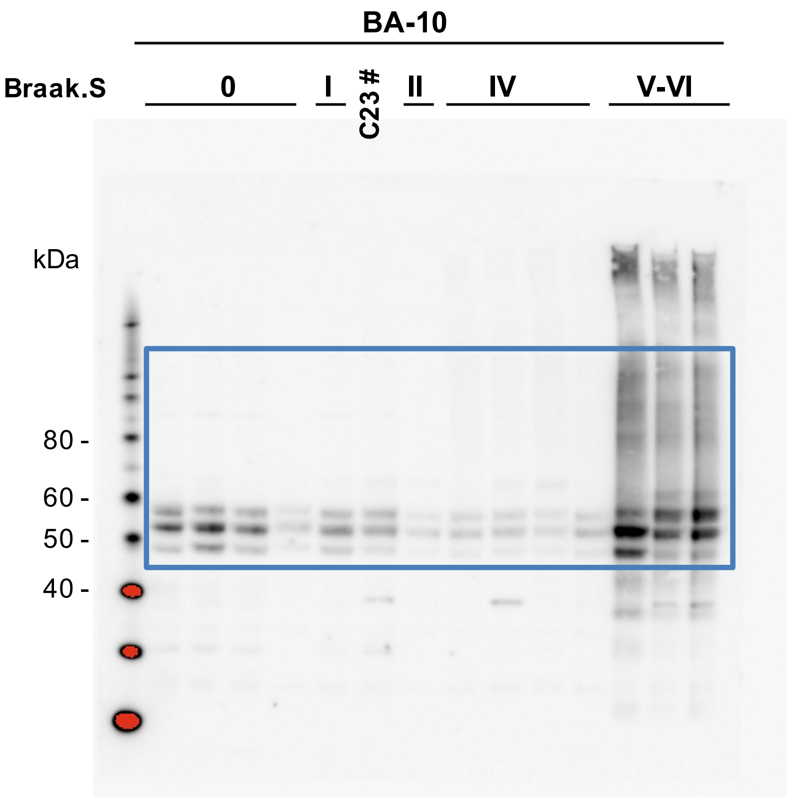

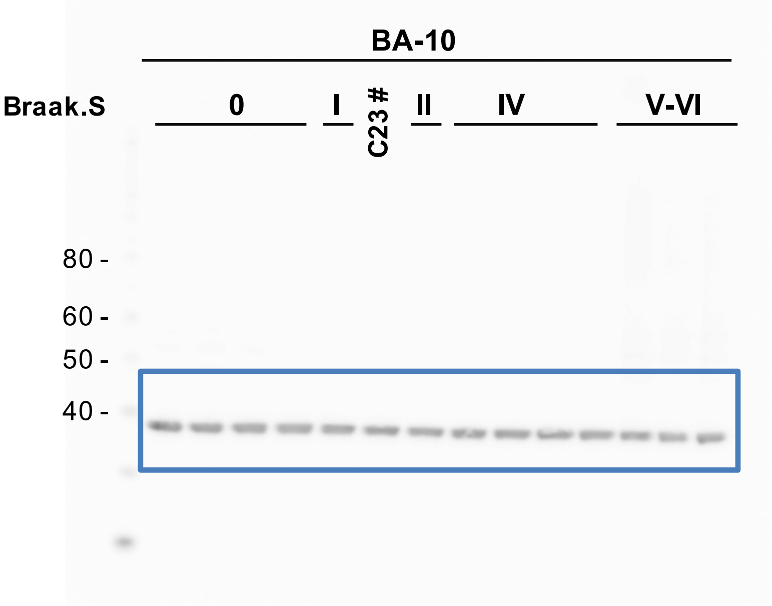

Fig. S4

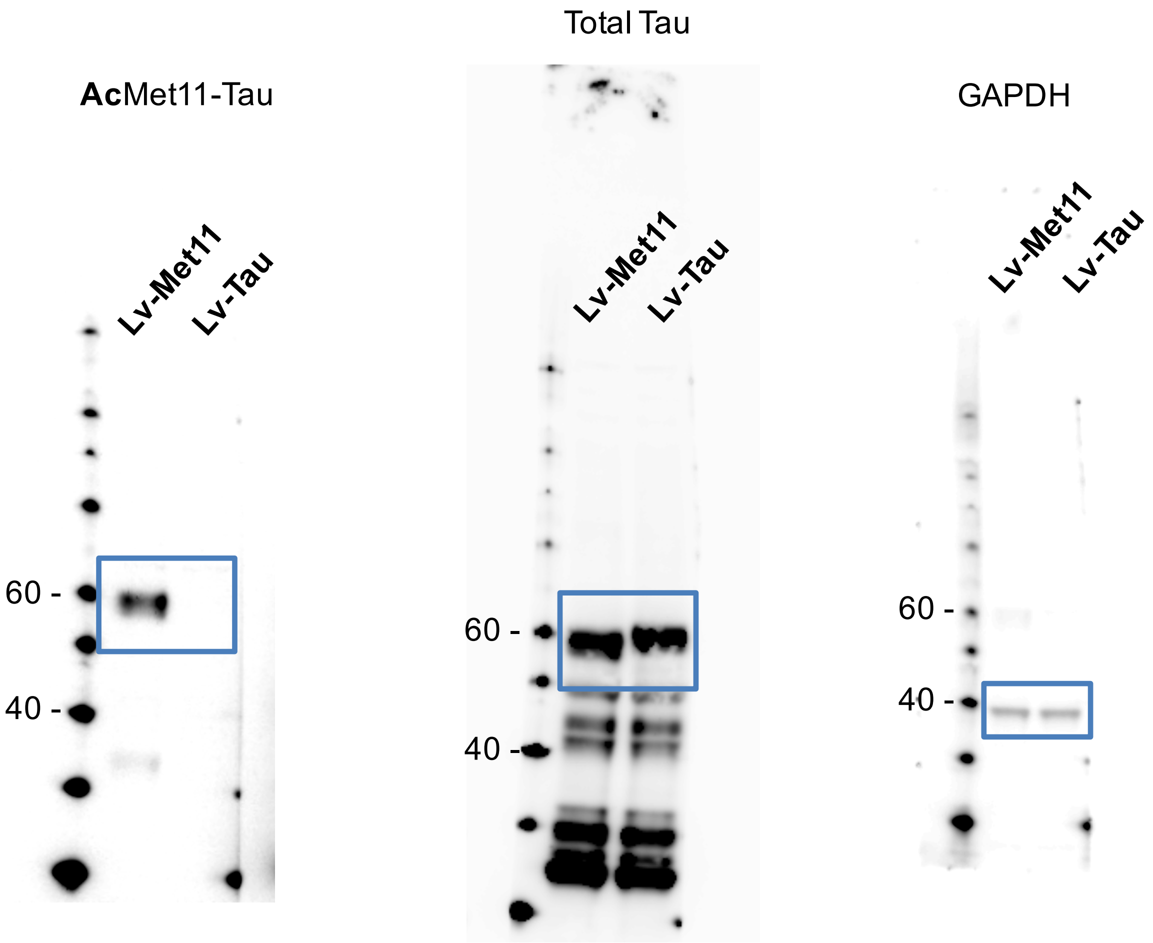
Total Tau
AcMet11-Tau
GAPDH
Lv-Met11
Lv-Tau
Lv-Met11
Lv-Tau
Lv-Met11
Lv-Tau
60 -
40 -
60 -
40 -
60 -
40 -
